# ComBatLS: A Location‐ and Scale‐Preserving Method for Multi‐Site Image Harmonization

**DOI:** 10.1002/hbm.70197

**Published:** 2025-06-11

**Authors:** Margaret Gardner, Russell T. Shinohara, Richard A. I. Bethlehem, Rafael Romero‐Garcia, Varun Warrier, Lena Dorfschmidt, Lifespan Brain Chart Consortium, Sheila Shanmugan, Paul Thompson, Jakob Seidlitz, Aaron F. Alexander‐Bloch, Andrew A. Chen

**Affiliations:** ^1^ Brain‐Gene‐Development Lab The Children's Hospital of Philadelphia and Penn Medicine Philadelphia Pennsylvania USA; ^2^ Neuroscience Graduate Group Perelman School of Medicine, University of Pennsylvania Philadelphia Pennsylvania USA; ^3^ Penn Statistics in Imaging and Visualization Center, Department of Biostatistics, Epidemiology, and Informatics University of Pennsylvania, Perelman School of Medicine Philadelphia Pennsylvania USA; ^4^ Center for Biomedical Imaging Computing and Analytics University of Pennsylvania, Perelman School of Medicine Philadelphia USA; ^5^ Department of Psychology University of Cambridge Cambridge UK; ^6^ Instituto de Biomedicina de Sevilla (IBiS) HUVR/CSIC/Universidad de Sevilla/CIBERSAM, ISCIII, Dpto de Fisiología Médica y Biofísica Barcelona Spain; ^7^ Department of Psychiatry University of Cambridge Cambridge UK; ^8^ Lifespan Brain Institute The Children's Hospital of Philadelphia and Penn Medicine Philadelphia Pennsylvania USA; ^9^ Department of Psychiatry University of Pennsylvania Philadelphia Pennsylvania USA; ^10^ Penn Lifespan Informatics and Neuroimaging Center University of Pennsylvania Philadelphia Pennsylvania USA; ^11^ Imaging Genetics Center Stevens Institute for Neuroimaging & Informatics, Keck School of Medicine, University of Southern California Los Angeles California USA; ^12^ Department of Child and Adolescent Psychiatry and Behavioral Science The Children's Hospital of Philadelphia Philadelphia Pennsylvania USA; ^13^ Department of Public Health Sciences Medical University of South Carolina Charleston South Carolina USA

## Abstract

Recent study has leveraged massive datasets and advanced harmonization methods to construct normative models of neuroanatomical features and benchmark individuals' morphology. However, current harmonization tools do not preserve the effects of biological covariates including sex and age on features' variances; this failure may induce error in normative scores, particularly when such factors are distributed unequally across sites. Here, we introduce a new extension of the popular ComBat harmonization method, ComBatLS, that preserves biological variance in features' locations and scales. We use UK Biobank data to show that ComBatLS robustly replicates individuals' normative scores better than other ComBat methods when subjects are assigned to sex‐imbalanced synthetic “sites.” Additionally, we demonstrate that ComBatLS significantly reduces sex biases in normative scores compared to traditional methods. Finally, we show that ComBatLS successfully harmonizes consortium data collected across over 50 studies. R implementation of ComBatLS is available at https://github.com/andy1764/ComBatFamily.

## Graphical abstract

We introduce ComBatLS, a new harmonization method that preserves the effects of biological covariates like sex and age on brain feature distributions' variances or scale parameters. We show that ComBatLS harmonization produces more accurate normative models and individual scores than other methods while removing batch effects.

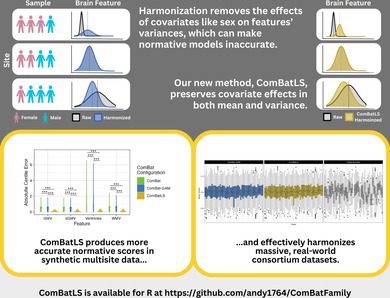

## 1 Introduction

Neuroimaging research's recent shift towards large sample sizes necessitates pooling data collected across many sites and scanners (Marek et al. 2022; Smith and Nichols 2018; Thompson et al. 2020). Both large consortia and multisite studies often use statistical methods to correct batch effects resulting from subtle differences in magnetic resonance imaging (MRI) fund acquisition, hardware, or collection protocols across sites (Bayer, Thompson et al. 2022; Hu et al. 2023). One popular technique is ComBat (Johnson et al. 2007), which estimates and removes site‐specific offsets in feature distributions while preserving the linear effects of prespecified covariates (J. P. Fortin et al. 2018; J.‐P. Fortin et al. 2017). This framework has been extended to create several new methods, including ComBat‐GAM, which preserves nonlinear covariate effects via generalized additive models (Pomponio et al. 2020) CovBat, which removes site effects in feature covariance (Chen et al. 2022); and DeepComBat, which leverages machine learning to perform multivariate harmonization across features (Hu et al. 2024). Collectively, these methods perform well in large samples across a range of imaging modalities (Orlhac et al. 2022; M. Yu et al. 2018) and increase statistical power (Radua et al. 2020; Sun et al. 2022). However, while they preserve covariate effects on features' means, no existing harmonization methods preserve the effects of biological covariates on neuroanatomical features' variances.

These biological sources of variance are integral to brain phenotypes' distributions across a population. Recent literature has demonstrated that factors such as age and biological sex impact the variances of neuroanatomical features (Dickie et al. 2013; Forde et al. 2020; Lange et al. 1997; Wierenga et al. 2018, 2022). While mean differences have received greater attention, sex differences in brain structures' inter‐individual variabilities are common and may impact disease prevalence (Forde et al. 2020; Wierenga et al. 2018, 2022; Williams et al. 2021). Similarly, assessments in adolescents (Lange et al. 1997), elderly populations (Dickie et al. 2013), and across the lifespan (Bethlehem et al. 2022; Dima et al. 2021) have found age‐related differences in the variances of several neuroanatomical features.

Biologically valid models of features' scales, or distributional dispersions, and robust harmonization are critical for generating normative trajectories of neuroanatomical features. This longstanding goal of neuroimaging research has recently become feasible thanks to massive consortia and open‐access datasets (Bethlehem et al. 2022; Dima et al. 2021; Frangou et al. 2022; Rutherford et al. 2023). Besides mapping a normative trajectory of brain structure throughout life, these models make it possible to quantify each individual's offset from this norm as a percentile (centile) or z‐score. Crucially, these scores depend on accurate models of population‐level variance accounting for factors like age and sex, making them highly susceptible to the loss of biological variability imposed by current harmonization methods.

Existing techniques' failure to preserve biological scale effects may be particularly problematic when such variance‐altering phenotypes are distributed unequally across sites (Nygaard et al. 2016). While some multisite studies are careful to maintain the same sample demographics across sites, others vary substantially in factors like sex, age, or diagnosis (see Data S1). Demographic imbalance becomes even more likely when data are compiled for secondary analyses, as is often the case for normative models. For example, current ComBat methods would force sites with male‐dominated and female‐dominated samples to target the same variance despite known sex differences in features' scales (J.‐P. Fortin et al. 2017). While this may be avoided by harmonizing males and females separately, this comes at the expense of statistical power and is not possible for continuous covariates like age. Thus, by removing biological effects on feature variance, harmonization may produce inaccurate or biased estimates of normative scores, which in turn could yield inaccurate or biased associations with phenotypes such as clinical diagnosis. Therefore, preserving the effects of covariates like age and sex on feature variance is crucial to estimating accurate normative scores for all individuals.

In this study, we extend the ComBat framework to perform batch correction while flexibly preserving covariates' effects on each feature's location and scale. This new method, ComBatLS, integrates generalized additive models for location, scale, and shape (GAMLSS) (Rigby and Stasinopoulos 2005) to preserve complex, nonlinear effects in both the first and second order of a feature's distribution during harmonization. We first assess ComBatLS's ability to preserve covariate effects on scale by applying it to synthetic “sites” containing unequal numbers of males and females, which we created from UK Biobank data (Littlejohns et al. 2020). Here, we focus on sex since it is commonly imbalanced in biomedical research samples (Bach et al. 2015; Dickinson et al. 2012; Rechlin et al. 2022) and its effects on neuroanatomical variance are well documented, including in this sample (Ritchie et al. 2018; Williams et al. 2021); however, we note that the same logic underlying ComBatLS is generalizable to any non‐technical covariate impacting neuroanatomical scale, much as other ComBat methods are able to preserve mean effects from any designated covariates (Johnson et al. 2007). We hypothesized that normative scores calculated from data harmonized across synthetic sites with ComBatLS would recapitulate subjects' true scores more accurately than those derived from data harmonized with other ComBat methods. We also conducted several tests for sex biases in how each method impacts score estimates, hypothesizing that ComBatLS would reduce sex‐related biases more effectively than other ComBat methods. Finally, we validated that ComBatLS successfully harmonizes massive datasets with varying demographics by applying it to data from the Lifespan Brain Chart Consortium (LBCC) (Bethlehem et al. 2022) collected by over 50 primary studies. We have released ComBatLS as part of the ComBat family of R methods at https://github.com/andy1764/ComBatFamily (Chen and Gardner 2024).

## 2 Methods

### 2.1 ComBatLS

CombatLS extends the original ComBat framework by flexibly modeling and preserving the effects of specified covariates on each feature's location and scale. ComBatLS directly models the first and second moments of each feature's distribution using generalized additive model for location, scale, and shape (GAMLSS) (Rigby and Stasinopoulos 2005) before shrinking site parameter estimates toward their mean estimate across features, thus preserving nonlinear covariate effects in both mean and variance while removing batch effects. By modeling covariate effects via GAMLSS, ComBatLS extends ComBat‐GAM (Pomponio et al. 2020). Specifically, ComBat‐GAM assumes that for site *i*, subject *j*, and feature *k*, $e_{\mathrm{ijk}}\sim N\left(0,{\sigma^{2}}_{k}\right)$ in

$$y_{\mathrm{ijk}}=\alpha_{k}+f_{k}\left({x^{T}}_{\mathrm{ij}}\right)+\gamma_{\mathrm{ik}}+\delta_{\mathrm{ik}}e_{\mathrm{ijk}}$$

In ComBatLS, we modify our model to incorporate a log‐linear relationship (Harvey 1976) between the error standard deviation and the covariates.

$$e_{\mathrm{ijk}}\sim N\left(0,{\sigma^{2}}_{\mathrm{ik}}\right)\;\;\log\left(\sigma_{\mathrm{ijk}}\right)=\zeta_{k}+{x^{T}}_{\mathrm{ij}}\eta_{k}$$

We assume a log‐linear relationship to reduce the risk of overfitting and ensure positive variance estimates, though other link functions may also be chosen (Harvey 1976; Stasinopoulos and Rigby 2008). Denote the standard data as $z_{\mathrm{ijk}}=\frac{y_{\mathrm{ijk}}-{\hat{\alpha }}_{k}+f_{k}\left({x^{T}}_{\mathrm{ij}}\right)}{\;{\hat{\sigma }}_{\mathrm{ik}}}=\frac{y_{\mathrm{ijk}}-{\hat{\alpha }}_{k}+f_{k}\left({x^{T}}_{\mathrm{ij}}\right)}{\;\sqrt{\exp\left({\hat{\zeta }}_{k}+{x^{T}}_{\mathrm{ij}}\hat{\eta_{k}}\right)}}$. We then obtain our harmonized observations as

$${y_{\mathrm{ijk}}}^{\text{ComBatLS}}=\frac{\sqrt{\exp\left({\hat{\zeta }}_{k}+{x^{T}}_{\mathrm{ij}}\hat{\eta_{k}}\right)}}{\delta {*}_{\mathrm{ik}}}\left(z_{\mathrm{ijk}}-{\gamma^{*}}_{\mathrm{ik}}\right)+{\hat{\alpha }}_{k}+f_{k}\left(\;{x^{T}}_{\mathrm{ij}}\right)$$

An important consideration arises in how to regress out site during the standardization step. Johnson, Li and Rabinovic (Johnson et al. 2007) obtain least‐squares estimates ${\hat{\gamma }}_{\mathrm{ik}}$ by constraining $\sum_{i}n_{i}{\hat{\gamma }}_{\mathrm{ik}}=0$ for all k=1,2,…,p. In ComBatLS, the log‐linear model of the error's standard deviation and covariates is estimated from the site‐influenced errors $\delta_{\mathrm{ik}}e_{\mathrm{ijk}}\sim N\left(0,{\delta^{2}}_{\mathrm{ik}}{\sigma^{2}}_{\mathrm{ik}}\right)$, which implies

$$\log\left(\delta_{\mathrm{ik}}\sigma_{\mathrm{ik}}\right)=\zeta_{k}+{x^{T}}_{\mathrm{ij}}\eta_{k}+\log\left(\delta_{\mathrm{ik}}\right)$$

For this to be identifiable (J.‐P. Fortin et al. 2017; Johnson et al. 2007), we first fit the model without an intercept to obtain estimates $\underline{\eta_{k}}$ and ${\underline{\sigma }}_{k}$. Then, we estimate the intercept as the pooled mean ${\hat{\zeta }}_{k}=\sum_{i}\frac{n_{i}}{n}\log\left({\underline{\delta }}_{\mathrm{ik}}\right)$. Finally, our estimates ${\hat{\delta }}_{\mathrm{ik}}$ are obtained as deviations from the estimated grand means ${\hat{\zeta }}_{k}$ via ${\hat{\delta }}_{\mathrm{ik}}={\underline{\delta }}_{\mathrm{ik}}/\exp\left({\hat{\zeta }}_{k}\right)$. Importantly, ComBatLS retains the strengths of prior ComBat iterations, including empirical Bayes methods to improve batch effect estimates for small sites (J.‐P. Fortin et al. 2017; Johnson et al. 2007). Briefly, this assumes normal and inverse‐gamma distributions as the priors for mean (${\gamma^{*}}_{\mathrm{ik}}$) and variance batch effects ($\delta {*}_{\mathrm{ik}}$), respectively. Hyperparameters are estimated via the empirical Bayes framework and used to compute conditional posterior estimates of location and scale effects (see Johnson et al. 2007 for details). Similarly, users can interrogate models' diagnostic plots using the plot.comfam() wrapper. See Hu et al. (Hu et al. 2023) for recommendations on assessing harmonization performance.

#### 2.1.1 Software

All code used to perform analyses and create figures is available at https://github.com/BGDlab/combat‐biovar. ComBatLS can be found at https://github.com/andy1764/ComBatFamily. Subject sampling, main analyses, and visualizations were done in R v 4.1.1. ComBat harmonization, sampling, and analyses for replications were done in R v 4.1.2.

### 2.2 Simulation Experiments to Demonstrate Covariate Preservation

#### 2.2.1 Overview

**FIGURE 1:**
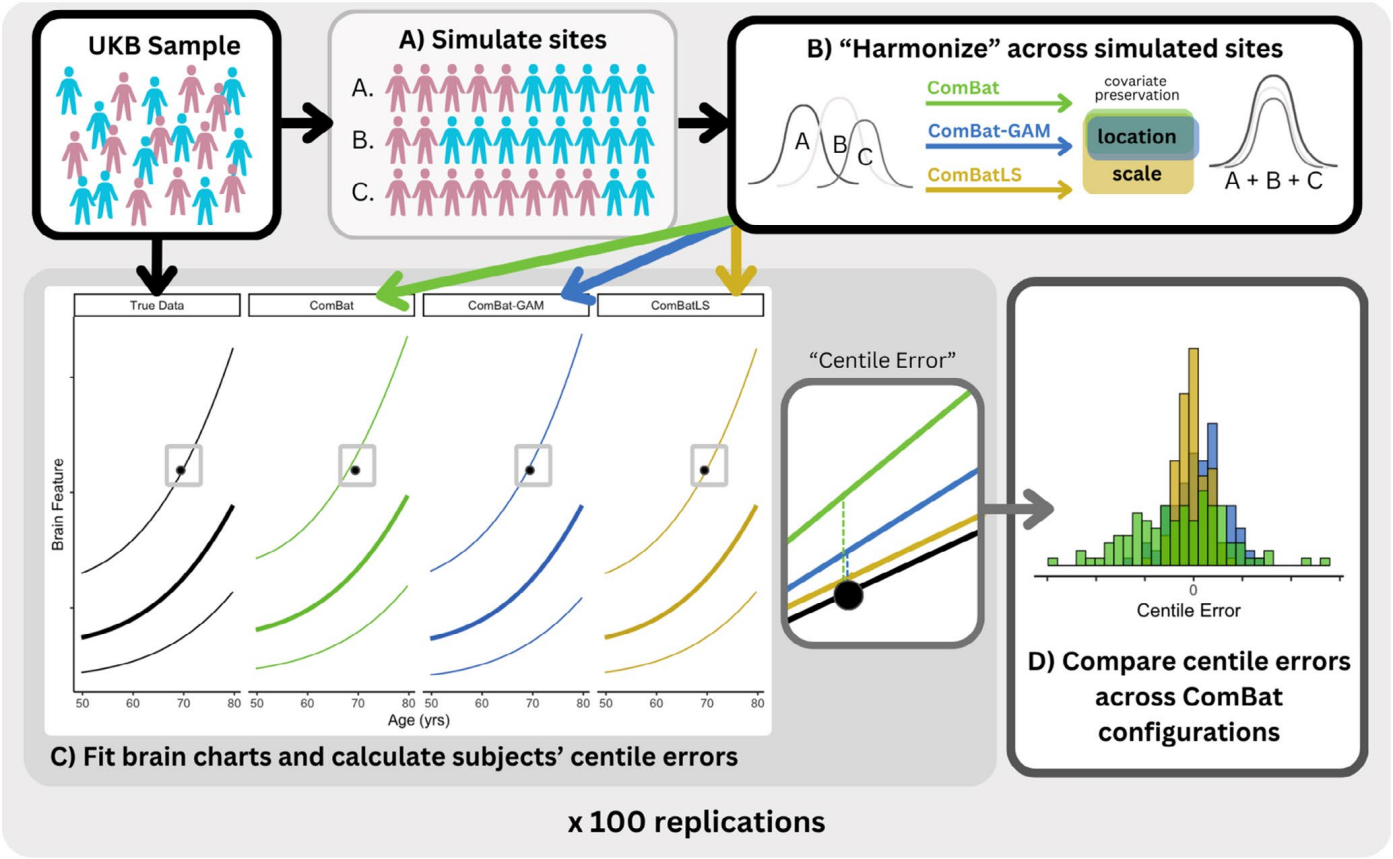
Summary of methodological approach for simulations. (A) Subjects from the UK Biobank (UKB) sample are randomly assigned to one of 3 simulated “study sites” such that sites have a male:female ratio of 1:1, 1:4, or 4:1. (B) Brain structure feature data are “harmonized” across these simulated sites using different configurations of ComBat, preserving covariates' effects on the mean (ComBatLS, ComBat‐GAM, linear ComBat) and/or variance (ComBatLS). (C) Brain growth charts are fit for brain features using the true structural data and data harmonized by each ComBat pipeline, which are then used to calculate personalized centile scores describing each subject's percentile relative to the population distribution. Centile error, defined as the difference between a subject's centile score when benchmarked on a brain chart modeled on “true” data and one fit on ComBat‐harmonized data, was calculated for each brain feature across all subjects. Lines represent brain charts of the 75th, 50th, and 25th percentiles for the feature given age; the solid point represents a single subject's brain feature, which has a “true” centile of 75% but corresponds to different centile scores when data is harmonized. (D) We analyzed the distributions of centile errors within and between ComBat configurations to assess the degree to which each method preserved biological variability in the simulated sites, thus minimizing centile errors.

To assess ComBatLS's ability to preserve the biologically derived variance necessary for calculating centile scores, we randomly sampled data from the UK Biobank (*N* = 28,619, 49.7% female) to create three synthetic sites, two with unequal sex ratios (Figure 1). We used ComBatLS to “harmonize” neuroanatomical features across these synthetic sites while preserving the effects of age and sex. Then, we compared subjects' centile scores from brain charts fit on these data to true centiles derived from the unharmonized dataset. Notably, since the synthetic “sites” were randomly assigned, there were no technical effects for any harmonization method to remove. Thus, any differences in centiles would result entirely from each method's preservation of biological covariates, with better‐preserving methods producing centile scores that most closely match these “true” centiles. In particular, since centile scores are defined by the population's distribution, recapitulating these true centiles requires that harmonization preserve between‐site differences in variance resulting from the samples' imbalanced sex compositions. We quantified the offset of each subject's ComBat‐derived centile scores from their true centiles as “centile error,” with smaller errors indicating better variance preservation. We then compared the distributions of centile errors and their magnitudes when data were harmonized by ComBatLS and three other methods: linear ComBat, ComBat‐GAM, and ComBat without any covariate preservation. Related methods such as DeepComBat (Hu et al. 2024), CovBat (Chen et al. 2022), and hierarchical Bayesian modeling (Bayer, Dinga et al. 2022) similarly restrict covariates' preserved effects to feature locations and thus suffer the same conceptual limitations as the chosen comparison methods (see Discussion). Results from ComBat without any covariate preservation—a theoretical “lower bound” to harmonization—are presented in the (Section S1, Figure S4–S13). To account for the randomness in creating synthetic sites, we resampled subjects' site assignments and repeated these analyses 100 times, which produced highly consistent results across replications (Section S1).

#### 2.2.2 UK Biobank Data

Our analyses leveraged structural MRI from the UK Biobank (UKB), a large, deeply phenotyped population sample of adults. Details of neuroimaging data acquisition are available elsewhere (Littlejohns et al. 2020). Importantly, while data were collected across three scan sites, exhaustive measures were taken to harmonize acquisitions, including identical scanner hardware, software, and acquisition sequences, as well as comprehensive, standardized staff trainings (Littlejohns et al. 2020).

T1‐ and T2‐FLAIR weighted images were obtained from the UK BioBank portal (application 20,904) and processed with FreeSurfer 6.0.1. We incorporated all scans that were defined as “usable” by the UK Biobank's in‐house quality control (Littlejohns et al. 2020) and completed FreeSurfer processing within 20 h. “Recon‐all” processing included bias field correction, registration to stereotaxic space, intensity normalization, skull‐stripping, and segmentation. T2‐FLAIR images were used to improve pial surface reconstruction when available. A triangular surface tessellation fitted a deformable mesh model onto the white matter volume, providing grey‐white and pial surfaces with > 160,000 corresponding vertices registered to fsaverage standard space. Surface area, thickness, and volumetric features were obtained for each of 68 cortical regions (34 per hemisphere) in the Desikan‐Killiany atlas (Desikan et al. 2006) from the aparc.stats files output by the “recon‐all” pipeline. Global cerebrum tissue volumes were extracted from the aseg.stats files output by the recon‐all process: “Total cortical gray matter volume” for GMV; “Total cerebral white matter volume” for WMV; and “Subcortical gray matter volume” for sGMV (inclusive of thalamus, caudate nucleus, putamen, pallidum, hippocampus, amygdala, and nucleus accumbens area; https://freesurfer.net/fswiki/SubcorticalSegmentation).

Analyses were restricted to individuals who had never had mental health problems as diagnosed by a mental health professional, per their response recorded in data‐field 20,544 (https://biobank.ndph.ox.ac.uk/showcase/field.cgi?id=20544) of the UKB mental health questionnaire. We further restricted the age range of our sample to subjects aged 50 to 80 years post‐conception, removing 411 individuals. This was done to reduce the possibility that inadequate representation of both males and females at each end of the sample would induce age differences between the synthetic sites.

#### 2.2.3 Synthetic Site Assignments

##### 2.2.3.1 Main Analyses

For our main analyses, each UKB subject in our sample was assigned to one of three synthetic sites using random sampling without replacement, in which the probability of assignment to a given site depended on sex. Female subjects were given a 33%, 58.75%, and 8.25% chance of being assigned to sites A, B, and C, respectively; male subjects had a 33%, 8.25%, and 58.75% likelihood of assignment across these same three sites. Thus, the sampling was constructed to create artificial sites of roughly equivalent size but with male:female ratios of 1:1 in site A, 1:4 in site B, and 4:1 in site C. This sampling was permuted 100 times for our replication analyses.

##### 2.2.3.2 Varying Male:Female Ratios

In addition, we assessed how the relative performance of ComBat methods varied with the imbalance in sites' male:female ratio by reassigning subjects to one of two synthetic sites: one with an equal number of males and females, and one with a sex ratio that varied across 11 permutations. Each site was limited to *N* = 9400 subjects, so that the 14,213 female UKB participants, who were a slight minority in the sample, could be assigned to one sex‐balanced site and one entirely female site without replacement. We used R's slice_sample() function to assign the appropriate number of randomly selected males and females to each site, with the number of males assigned to the second site increasing by 940 (10% of the site's sample) from zero to 9400 over 11 permutations.

#### 2.2.4 ComBat Harmonization

For each sampling permutation, we harmonized across the synthetic sites, or batches, using 4 ComBat methods: ComBat, ComBat‐GAM, ComBatLS, and linear ComBat without any covariate preservation. Harmonization was performed in R using the ComBatFamily package (https://github.com/andy1764/ComBatFamily). ComBat was fit while accounting for linear effects of age and sex, while ComBat‐GAM was fit with a thin plate regression spline for age and a linear effect of sex. For ComBatLS, the first moment was fit with a penalized b‐spline for age and a linear effect of sex, and the second moment was fit with linear effects for both sex and age. Empirical Bayesian estimation was used within each category of features—global tissue volumes and regional cortical volumes, thicknesses, and surface areas—to improve site effect estimates.

#### 2.2.5 Centile Score Calculation

For our simulations in UKB, we fit simple brain chart models of each feature using GAMLSS such that: $f_{k}$() = Box‐Cox Cole‐Green(μ,σ,υ) with

$$\mu_{\mathrm{ijk}}=\alpha_{\mu ,1}\mathrm{ag}e_{\mathrm{ij}}+\alpha_{\mu ,2}\mathrm{ag}{e_{\mathrm{ij}}}^{2}+\alpha_{\mu ,3}\mathrm{ag}{e_{\mathrm{ij}}}^{3}+\beta_{\mu }\mathrm{se}x_{\mathrm{ij}}+\zeta_{\mathrm{ijk}}$$

$$\log\left(\sigma_{\mathrm{ijk}}\right)=\alpha_{\sigma ,1}\mathrm{ag}e_{\mathrm{ij}}+\alpha_{\sigma ,2}\mathrm{ag}{e_{\mathrm{ij}}}^{2}+\alpha_{\sigma ,3}\mathrm{ag}{e_{\mathrm{ij}}}^{3}+\beta_{\sigma }\;\mathrm{se}x_{\mathrm{ij}}+\delta_{\mathrm{ijk}}$$

$$\nu_{\mathrm{ijk}}=\omega_{\mathrm{ijk}}$$

As above, age is calculated in days post‐conception, and sex is binarized with the reference level as “female.” We chose the default Box‐Cox Cole–Green distribution method to allow for z‐score estimation (see Section S1).

This model was fit on every feature's true, unharmonized distribution and on data harmonized with each ComBat method using the *gamlss* package. For each model, we obtained subjects' centile scores using the *predictAll()* function. This resulted in centile scores for each subject on all features' true values, as well as their values when harmonized by the four ComBat methods.

#### 2.2.6 Evaluating Relative Accuracy Across ComBat Methods

To assess the ability of each ComBat method to preserve biologically relevant sources of interindividual variability, even when they are distributed unequally across sites, we tested how closely subjects' centile scores matched “ground‐truth” centiles derived from unharmonized data. This within‐subject accuracy was quantified by subtracting the ground‐truth centile from the centile derived from harmonized data for each feature, referred to as “centile error.” Negative centile scores indicate that the ComBat method caused an underestimation of the centile score, while positive scores indicate ComBat‐induced centile inflation.

As our primary goal was to assess the overall efficacy of each harmonization method, we evaluated the magnitude of inaccuracy within each feature using pairwise comparisons of the absolute values of centile errors between methods. As these distributions were paired, skewed, and unequal in variance, we converted the absolute centile errors into ranks before conducting paired, two‐tailed *t*‐tests with Welch's correction (Ruxton 2006; Zimmerman and Zumbo 1993). Significantly smaller absolute centile errors indicated that a ComBat method preserved covariate effects in that feature more accurately than the ComBat method with larger absolute centile errors. Within each sampling replication, we applied FDR correction across 6 pairings of the four ComBat methods and 208 features for a total of 1248 comparisons. We applied these same methods to evaluate ComBat methods' accuracy in our 11 samples with varying male:female ratios, using FDR correction to account for 1248 comparisons * 11 ratios simulated.

#### 2.2.7 Assessing Sex Biases in Centile Scores Induced by ComBat Methods

We first visualized the effect of sex for each feature's scale. We used the drop1() function to assess the significance of the sex term in sigma for each feature's brain charts, as recommended (Stasinopoulos and Rigby 2008). We then defined the effect of sex on variance as the difference in variance predicted for a male minus the variance predicted for a female at the average age of the sample, 64.94 years post‐conception. We calculated a standardized effect of sex that would be comparable across brain features by dividing this sex effect by the predicted variance for females (the reference level for sex).

To determine whether ComBat harmonization differentially affected males and females when applied to sites with sex‐imbalanced samples, we conducted within‐feature, two‐tailed *t*‐tests of ranks with Welch's correction (Ruxton 2006; Zimmerman and Zumbo 1993) comparing males' and female's centile errors. These analyses were repeated across our 100 sampling replications as well as 11 simulated male:female ratios. We defined the size of this sex effect within a feature as the male centile error's median minus the female centile error's median, which produces a single sex difference metric.

We conducted further analyses to determine whether sex differences were systematic across features such that centile scores would be biased in a particular direction.

First, we summarized sex differences within each feature type—global tissue volumes, regional cortical volumes, regional cortical thickness, and regional surface area—by taking the median of their sex‐difference metrics. We repeated this procedure across our 100 sampling replications to create a distribution of median sex differences for each ComBat method and feature type, then used one‐sample *t*‐tests to assess whether these distributions' means differed from zero. A non‐zero mean would indicate that, for a given feature type, that ComBat method tends to bias centile scores by sex, with more negative distributions indicating that males' centiles tend to be underestimated relative to females'.

Next, we determined whether ComBat‐induced errors led to one sex being overrepresented among those with extreme centile scores, which could mask or skew associations between centiles and other phenotypes of interest. We first calculated subjects' average centile scores within global tissue volume, regional cortical volume, regional cortical thickness, and regional surface area features. We considered average centiles below 20% as “low” and above 80% as “high.” We used centiles derived from the true, unharmonized data to calculate what proportion of individuals in each category was expected to be female. Finally, we compared this expected proportion to the distribution of proportions calculated across 100 replications of harmonized data using two‐sided, one‐sample *t*‐tests.

Finally, we assessed directional sex biases across our 11 sampling permutations with varying sex ratios by calculating the sex difference in medians for each feature's centile errors, as in our main analyses. We first used within‐feature, two‐tailed *t*‐tests of ranks with Welch's correction to compare males' and females' centile errors, as above. We then used one‐sample *t*‐tests to assess whether the mean of these within‐feature sex differences differed from zero in any of the sex‐imbalanced permutations tested.

### 2.3 Harmonization of Neuroanatomical Features From the LBCC Dataset

#### 2.3.1 Overview

To assess its utility in real‐world samples, we applied ComBatLS and ComBat‐GAM to a large consortium sample of healthy subjects (Bethlehem et al. 2022). We then measured the relative performance of each harmonization method by assessing the magnitude of residual site effects. We further explored how often the methods diverged in their classifications of “atypical” centiles and whether these centile score differences are related to primary studies' demographics. As there are many ways to derive growth curves, we assessed ComBatLS's impact on normative models and scores as described later, as well as when employing a popular modeling strategy that has been used in other consortia (Dima et al. 2021; Frangou et al. 2022) (see Section S1).

#### 2.3.2 Lifespan Brain Chart Consortium Sample

The Lifespan Brain Chart Consortium (LBCC) is an aggregated collection of structural MRI scans, including data from individuals across the range of the human lifespan and the globe. Details of the dataset and primary studies, including diagnostic criteria, can be found elsewhere (Bethlehem et al. 2022). For these analyses, we used data from healthy individuals collected by 60 studies. We first excluded all scans collected before 3 years of age (*n* = 1693 scans), then randomly selected a single scan to retain from subjects with more than one timepoint, which removed a further 13,387 scans across 6566 individuals. We then assessed image quality using the Euler index, an automated measure of the reconstructions' surface continuity often used as a robust, quantitative assessment of scan quality (Rosen et al. 2018). As in prior work (Bethlehem et al. 2022), we applied an adaptive threshold of the Euler index, excluding scans with Euler indices greater than two median absolute deviations above the primary‐study median. This quality threshold removed a total of 6443 scans (8.00% of a study's primary sample on average), while a further 2062 subjects with missing values were removed via listwise deletion. Finally, three subjects with biologically implausible phenotypes suggestive of technical error (values > 20 median absolute deviations from the feature's overall median) were removed. Following quality control procedures, a total of 55,237 scans from 51 studies and 202 sites remained for analysis.

#### 2.3.3 ComBat Harmonization

We applied ComBat‐GAM and ComBatLS to harmonize this curated LBCC sample, treating primary collection studies as batches. Harmonization was performed on log‐transformed feature values to prevent ComBat from estimating negative feature values. We preserved the effects of age, sex, and their interaction by estimating as follows: study *i*, subject *j*, and feature *k*:

$$\text{ComBat}-\mathrm{GAM}:\mu_{\mathrm{ijk}}=s\left(\alpha_{\mathrm{age}}\mathrm{ag}e_{\mathrm{ij}}\right)+\beta_{\mathrm{sex}}\mathrm{se}x_{\mathrm{ij}}+\gamma_{\mathrm{int}}\left(\mathrm{ag}e_{\mathrm{ij}}\times \mathrm{se}x_{\mathrm{ij}}\right)+\zeta_{\mathrm{ijk},}$$

where s(.) signifies thin plate regression splines.

ComBatLS: Normal $\left(\mu ,\sigma \right)$ with

$$\mu_{\mathrm{ijk}}=\mathrm{pb}\left(\alpha_{\mu ,\mathrm{age}}\mathrm{ag}e_{\mathrm{ij}}\right)+\beta_{\mu ,\mathrm{sex}}\mathrm{se}x_{\mathrm{ij}}+\gamma_{\mathrm{int}}\left(\mathrm{ag}e_{\mathrm{ij}}\times \mathrm{se}x_{\mathrm{ij}}\right)+\zeta_{\mathrm{ijk}}$$

$$\log\left(\sigma_{\mathrm{ijk}}\right)=\mathrm{pb}\left(\alpha_{\sigma ,\mathrm{age}}\mathrm{ag}e_{\mathrm{ij}}\right)+\beta_{\sigma ,\mathrm{sex}}\mathrm{se}x_{\mathrm{ij}}+\delta_{\mathrm{ijk},}$$

where pb(.) signifies penalized b‐splines, with 20 knots allowed in the mu term and 5 knots in sigma. Models that failed to converge using the *gamlss* package default RS() algorithm were attempted using CG() (Stasinopoulos and Rigby 2008).

Empirical Bayesian estimation was used within each category of cortical features—volumes, thickness, and surface area—to improve site effect estimates, while effects for each global tissue volume were estimated individually.

#### 2.3.4 Centile Score Calculation

We used ComBatLS and ComBat‐GAM harmonized datasets to estimate each feature's normative trajectory across the LBCC sample with the following GAMLSS model:

$f_{k}$ = Box‐Cox Cole‐Green(μ,σ,υ) with

$$\begin{array}{cc} \mu_{\mathrm{ijk}}= & \mathrm{pb}\left(\alpha_{\mu ,\mathrm{age}}\mathrm{ag}e_{\mathrm{ij}}\right)+\beta_{\mu ,\mathrm{sex}}\mathrm{se}x_{\mathrm{ij}}+\mathrm{pb}\left(\gamma_{\mu ,\mathrm{int}}\mathrm{ag}e_{\mathrm{ij}}\times \mathrm{se}x_{\mathrm{ij}}\right)\\ & +\eta_{\mu ,\mathrm{fs}}\text{freesurfe}r_{\mathrm{ij}}+\zeta_{\mathrm{ijk}} \end{array}$$

$$\begin{array}{cc} \log\left(\sigma_{\mathrm{ijk}}\right)= & \mathrm{pb}\left(\alpha_{\sigma ,\mathrm{age}}\mathrm{ag}e_{\mathrm{ij}}\right)+\beta_{\sigma ,\mathrm{sex}}\mathrm{se}x_{\mathrm{ij}}+\mathrm{pb}\left(\gamma_{\sigma ,\mathrm{int}}\mathrm{ag}e_{\mathrm{ij}}\times \mathrm{se}x_{\mathrm{ij}}\right)\\ & +\eta_{\sigma ,\mathrm{fs}}\text{freesurfe}r_{\mathrm{ij}}+\delta_{\mathrm{ijk}} \end{array}$$

$$\begin{array}{cc} \nu_{\mathrm{ijk}}= & \mathrm{pb}\left(\alpha_{\nu ,\mathrm{age}}\mathrm{ag}e_{\mathrm{ij}}\right)+\beta_{\nu ,\mathrm{sex}}\mathrm{se}x_{\mathrm{ij}}+\mathrm{pb}\left(\gamma_{\nu ,\mathrm{int}}\mathrm{ag}e_{\mathrm{ij}}\times \mathrm{se}x_{\mathrm{ij}}\right)\\ & +\eta_{\nu ,\mathrm{fs}}\text{freesurfe}r_{\mathrm{ij}}+\omega_{\mathrm{ijk}}, \end{array}$$

where pb(.) signifies penalized b‐splines. As above, age is calculated in days post‐conception, and sex is binarized with the reference level as “female.” We also controlled for variation in image‐processing software (“freesurfer”).

Subjects' centile scores were derived from each model using the predictAll() function. Models fit on unharmonized data were identical, except that we applied a log‐link function in μ to mirror the log‐scaling applied in LBCC harmonization (see Section 2.3.3).

#### 2.3.5 Site‐Effect Quantification

We conducted two tests to assess how well ComBatLS harmonizes real‐world data, using data harmonized by ComBat‐GAM as a “silver standard” and the raw data as a lower bound. To directly quantify the residual effects of primary study within each feature, we re‐fit the gamlss brain chart models specified above on the ComBatLS‐ and ComBat‐GAM‐harmonized datasets, this time including fixed effects of study in the mu and sigma moments. We fit these same models on the raw dataset with a log‐link function in μ. From there, we used generalized pseudo R‐squared (Nagelkerke 1991; Stasinopoulos and Rigby 2008) to calculate the local effect size of “study” via Cohen's F‐squared (Selya et al. 2012).

#### 2.3.6 Comparison of ComBatLS and ComBat‐GAM and Associations With Sites' Sample Characteristics

While no “ground truth” for harmonized LBCC data exists, we evaluated whether differences in subjects' centile scores when harmonized with ComBatLS and ComBat‐GAM were related to the distribution of biological sources of variability across studies. Specifically, as studies tended only contain subjects from a relatively narrow age range, we assessed whether a subject's average magnitude of difference in ComBatLS‐ and ComBat‐GAM‐derived centiles was associated with the mean age of its primary study, the range of ages encompassed in that primary study, or how greatly that individual deviated from their primary study's mean age. For each subject, we calculated differences in centiles from the two methods and took the mean of their absolute values across features. We also calculated the mean age of each site's sample and the absolute difference between that mean age and the ages of each individual in the study sample. Finally, we used linear regression to test subjects' mean absolute centile differences against the study's mean age, age range, and their absolute deviation from that mean age, while controlling for study sample size.

## 3 Results

### 3.1 ComBatLS Recapitulates Centile Scores Across Sex‐Imbalanced Sites Better Than Other ComBat Methods

**FIGURE 2:**
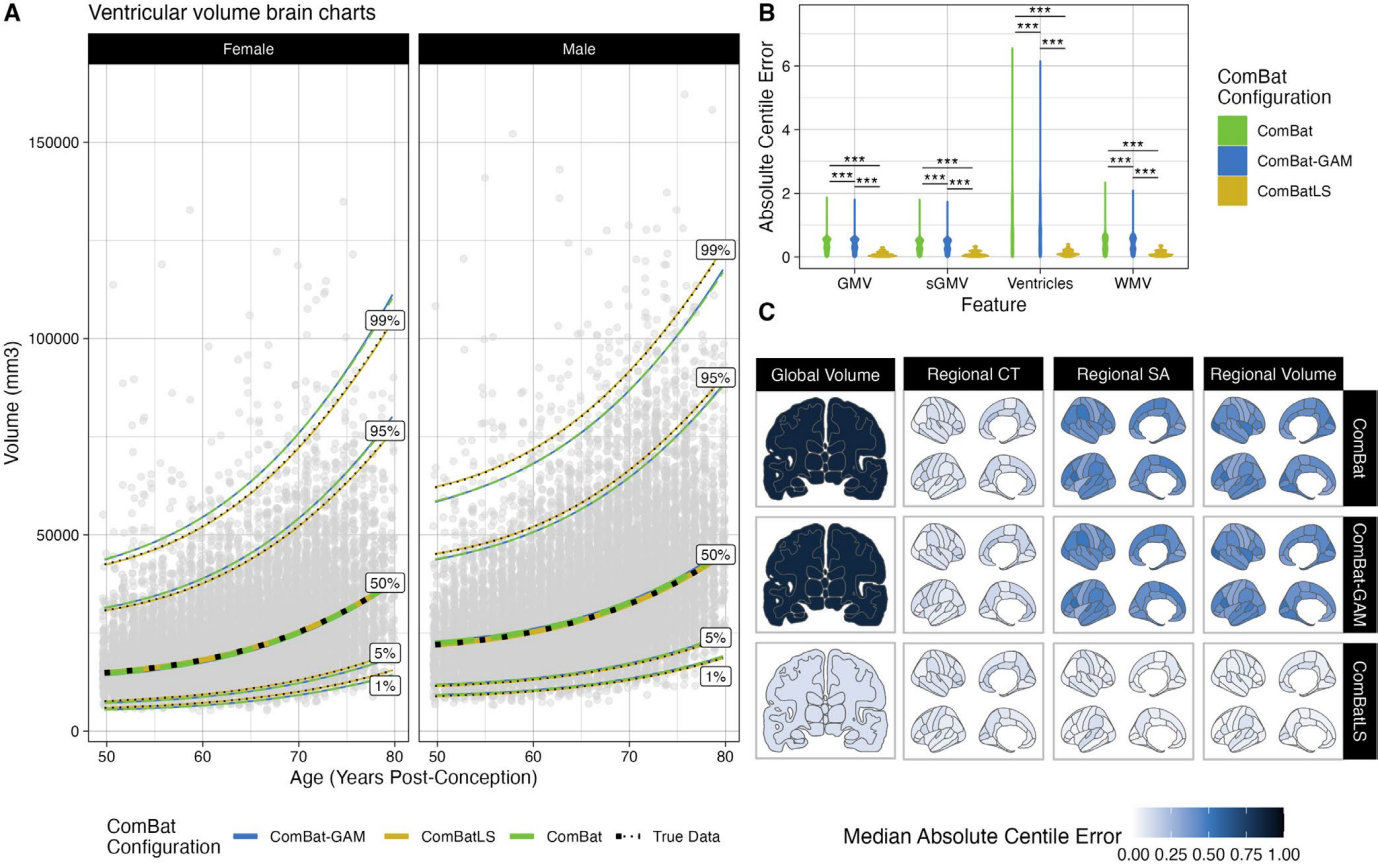
ComBatLS best replicates centile predictions across simulated sex‐imbalanced sites. (A) Brain charts of ventricular volume in females and males when derived from data harmonized across simulated sites with ComBat, ComBat‐GAM, or ComBatLS relative to unharmonized (“true”) data. The line color corresponds to ComBat configuration, black dotted line corresponds to true data. Age values jittered slightly for visualization. (B) Absolute centile errors, or distance in centile space between subjects' true centiles and centiles derived from data harmonized with different ComBat configurations, of global volumetric features. (C) Median absolute centile errors across brain features in data harmonized with varying ComBat configurations. Abbrv: WMV, white matter volume, GMV, cortical gray matter volume, sGMV, subcortical gray matter volume, CT, cortical thickness; SA, surface area; ***, *p* < 0.001, FDR‐corrected.

We first fit GAMLSS models to derive centile scores from 208 neuroanatomical features—four global tissue volumes and 68 cortical regions' volume, surface area, and thickness measures—harmonized by each ComBat method (Figure 2A, see Methods). Using non‐parametric, paired, two‐sample tests of absolute centile errors, we found that in nearly all features, ComBatLS had significantly lower absolute centile errors than linear ComBat or ComBat‐GAM (Figure 2B,C; Section S1: Table S1, Figure S4–S6), indicating that this method induces less error in centile scores when harmonizing across sex‐imbalanced simulated sites. Across our 100 replications, all volume and surface area features' absolute centile errors were lowest when fit with ComBatLS. However, a small number of cortical thickness features were fit better by ComBat‐GAM or ComBat (Figure S5), which both performed better in cortical thickness than other features. This is expected because, as in prior studies (Forde et al. 2020; Ritchie et al. 2018; Wierenga et al. 2018, 2022), the variance of cortical thickness is less impacted by sex than the variances of other features modeled in this sample (Section S1, Figure S14); therefore, less information is lost when harmonization does not preserve these effects. ComBatLS's added utility beyond other ComBat methods is thus proportional to the degree to which preserved covariates impact features' variances, though it performs well even in the absence of substantial scale effects. In sum, these results demonstrate that ComBatLS is highly effective in preserving biological covariates' effects on features' scales, even when those covariates are not distributed evenly across study sites.

In addition to replicating these analyses after re‐sampling subjects' site assignment 100 times (Table S1), we assessed whether extreme phenotypes drove the observed results by removing centile scores > 95% or < 5% when calculated on the original data. Again, these results were highly similar to those of the full dataset (Section S1, Figures S15–S17). We also repeated these analyses using Z‐scores (theoretical range (−inf, inf)) which yielded a nearly identical pattern of results to centile scores (range (0,1)) (Section S1, Figure S18–S28). Together, these analyses indicate that ComBatLS is consistent and robust to extreme phenotypes and the choice of normative score.

### 3.2 Sex Differences Induced by ComBatLS Are Small and Less Directionally Biased Than Other ComBat Methods

**FIGURE 3:**
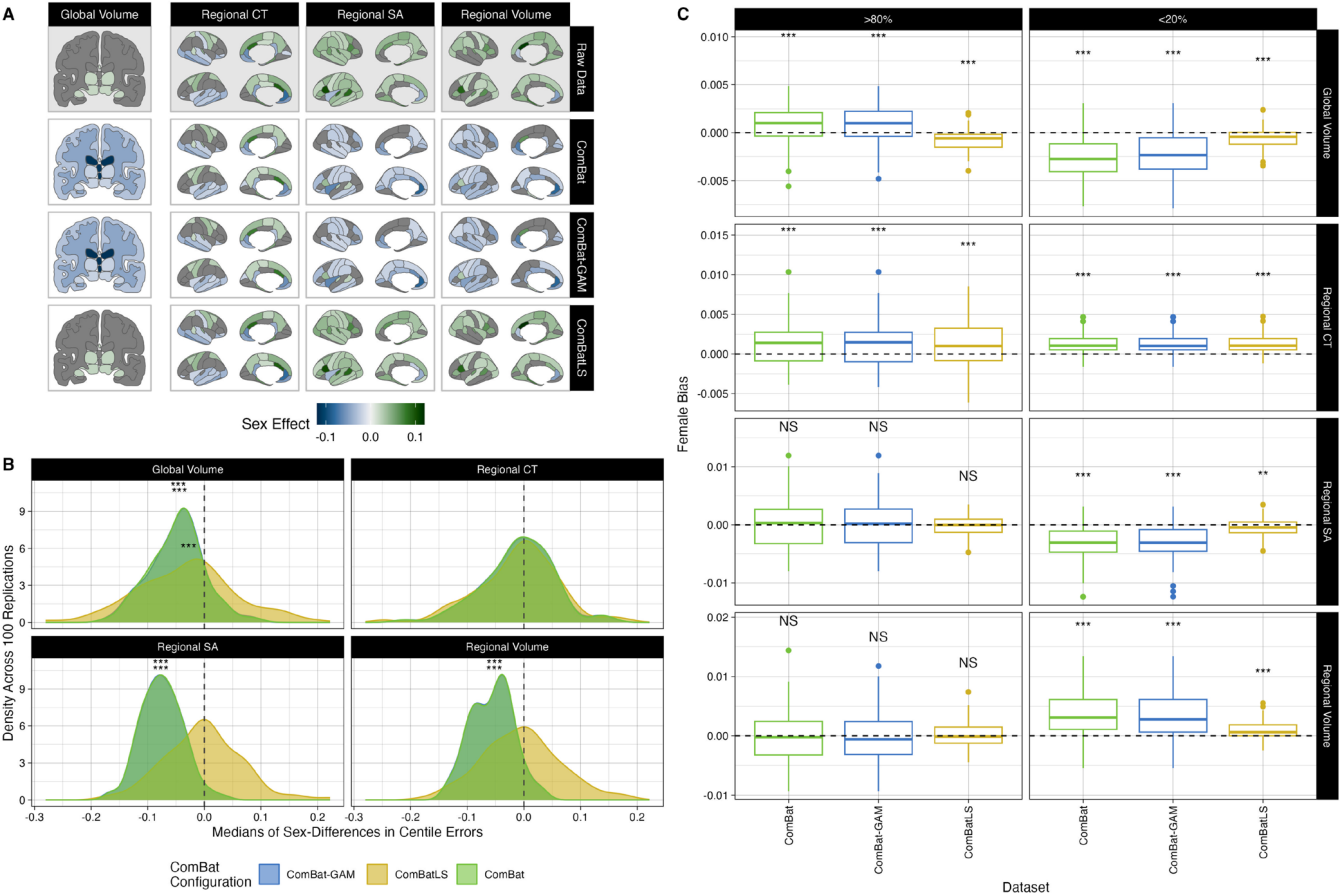
ComBatLS induces less sex bias in centile scores than other ComBat methods. (A) Brain features with significant sex effects in scale, as determined by the second moment of GAMLSS growth charts. Fill represents the difference in males' and females' predicted variance at the sample's mean age (64.94 years), standardized by dividing by females' predicted variance. Positive effects indicate that males' variance is higher than females'. Gray regions indicate non‐significant sex effects. (B) Density plots of median sex differences in centile errors induced by different ComBat methods within phenotype categories across 100 replications. (C) Bias in the proportion of females with low (< 20th percentile) or high (> 80th) mean centiles across 100 sampling replications. Positive values indicate a higher proportion of females than “true” mean centiles calculated from unharmonized data (dashed line). Boxplots indicate median proportion of females across replications, with hinges corresponding to 1st and 3rd quartiles and whiskers extending to the most distant value no farther than + − 1.5*IQR from the hinge. Abbrv: CT, cortical thickness; SA, surface area; ***, *p* < 0.001; **, *p* < 0.01, two‐sided, one‐sample *t*‐tests.

We next assessed whether the centile errors induced by each ComBat method vary by subject sex, producing centile estimates that could bias associations of interest. We first mapped significant effects of sex on variance estimated by each feature's brain charts, which revealed that models fit on ComBatLS‐harmonized synthetic data closely match effects quantified in the true data (Figure 3A). To see whether these discrepancies in sex estimates affect centile scores, we used Wilcoxon tests to compare males' and females' centile errors across ComBat methods within each feature. For all methods, most of the 208 features exhibited significant sex differences in centile errors (ComBatLS: 199 features, ComBat‐GAM: 142 features, ComBat: 141 features; Figure S8). Next, to test whether these differences produced sex biases by consistently over‐ or under‐estimating either sex's centiles, we examined the distributions of these sex differences' medians across 100 sampling replications. This revealed that linear ComBat and ComBat‐GAM tend to produce slightly more negative sex differences in centile errors, corresponding to a systematic underestimation of males' centile scores relative to females' (Figure 3B; Figures S9 and S10). ComBatLS produced a very small but significant negative male bias only in global tissue volume centiles (*β* = −3.1e‐04 centiles, *t* = −3.62, df = 99, *p* = 0.0005), while again, cortical thickness was exceptional in that significant biases were also absent in ComBat‐GAM and ComBat (ComBat‐GAM: *β* = −9.13e‐05 centiles, *t* = −1.52, df = 99, *p* = 0.130, ComBat: *β* = −9.57e‐05 centiles, *t* = −1.57, df = 99, *p* = 0.12). We further visualized whether these biases resulted in over‐representation of either sex among subjects with very high (> 80%) or very low (< 20%) average centiles in any feature category. Interestingly, we found that ComBatLS, ComBat‐GAM and ComBat all tend to slightly over‐ or under‐represent females among those with extreme average centiles (Figure 3C). These results did not change when FDR correction was applied to account for multiple comparisons. Together, these results suggest that ComBatLS‐harmonized data preserves sex's effects on variance and thus induces the smallest and least‐biasing differences in males' and females' centiles.

### 3.3 ComBatLS Preserves Covariate Effects Across a Wide Range of Sex Imbalances in Synthetic Sites

**FIGURE 4:**
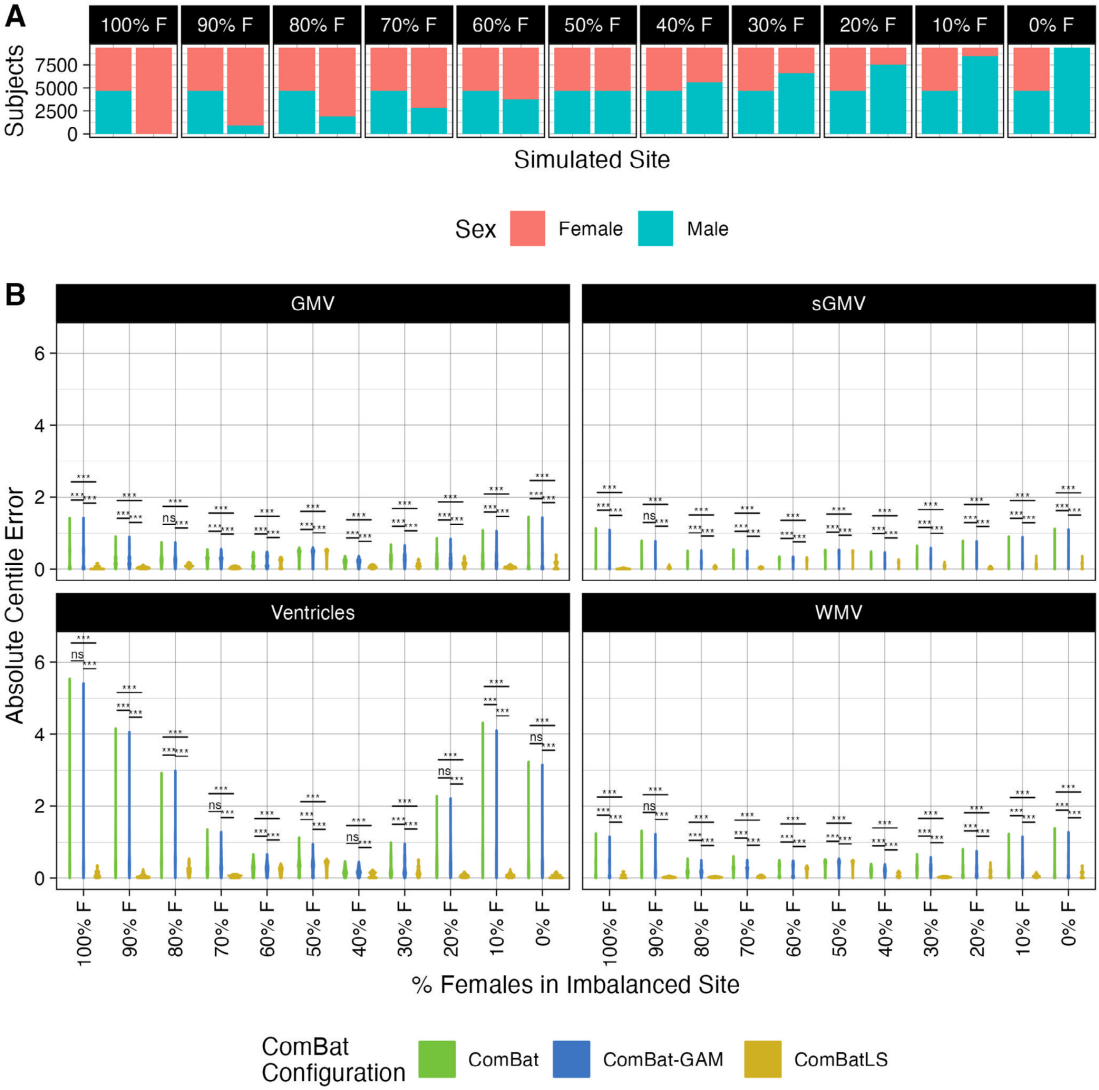
ComBatLS best preserves the effects of biological sex on centile scores across varying degrees of site‐level sex imbalance. (A) Distribution of female and male UKB subjects across two simulated sites, permuted 11 times to assess the performance of ComBat configurations across varying degrees of site‐level sex imbalance. (B) Distributions of absolute centile errors of global brain features harmonized across sites simulated with varying degrees of sex imbalance using ComBat, ComBat‐GAM, and ComBatLS. Abbrviations: CT, cortical thickness; GMV, cortical gray matter volume; SA, surface area; sGMV, subcortical gray matter volume; WMV, white matter volume, ***, *p* < 0.001; **, *p* < 0.01, FDR‐corrected.

To test how each ComBat method's performance is affected by varying degrees of covariate imbalances, we again drew eleven samplings of UK Biobank participants to create two synthetic sites: one with an even sex ratio and one in which the percentage of males ranged from 0% to 100% in 10% increments (Figure 4A). We then repeated the same procedures as in our main analyses to obtain and compare centile errors between ComBat methods when harmonizing each of these eleven samples.

For most features, ComBatLS produced significantly smaller absolute centile errors than other ComBat methods when one site was imbalanced for sex (Figure 4B). As above, these gains are less apparent in cortical thickness measures and when both sites are perfectly balanced for sex (Figure S12). Our assessment of sex biases in each method's centile errors was again consistent with our main analyses (Figure S13; Section S1).

These analyses demonstrate that ComBatLS adaptively preserves biological covariates' effects on variance in a range of simulations and becomes more beneficial to centile accuracy as samples' compositions become increasingly imbalanced.

### 3.4 ComBatLS Effectively Harmonizes Massive Real‐World Datasets

**FIGURE 5:**
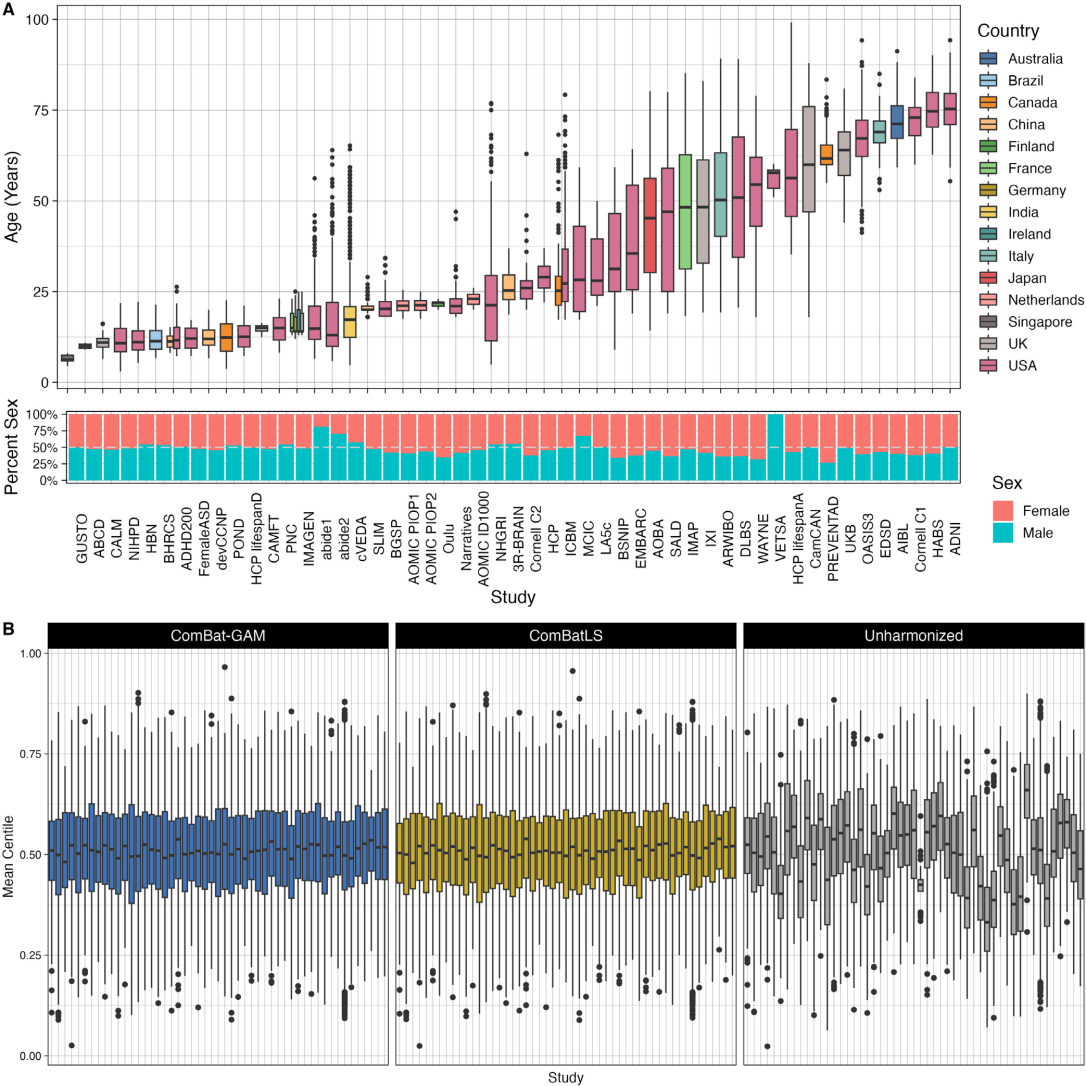
Both ComBatLS and ComBat‐GAM effectively remove batch effects from consortium data containing over 50 studies. (A) Characteristics of curated LBCC sample by primary study and summarized across the full sample. (B) Distributions of subjects' mean centiles across all brain features within each primary study. Centiles are calculated from brain charts fit on data harmonized using ComBat‐GAM or ComBatLS, or on unharmonized data, and averaged within subjects across features. Boxplot line indicates median with hinges corresponding to 1st and 3rd quartiles and whiskers extending to the most distant value no farther than + − 1.5*IQR from the hinge.

To assess its utility in real‐world samples, we applied ComBatLS and ComBat‐GAM to data from the Lifespan Brain Chart Consortium, a global sample of structural MRI from healthy individuals across the human lifespan (Bethlehem et al. 2022). Here we used scans from 55,237 (51.3% female) unique subjects aged 3.0–99.2 years collected by 51 primary studies over 202 sites (Figure 5A, see Methods for data curation). We first established ComBatLS' ability to harmonizes true batch effects. We found that both ComBatLS and ComBat‐GAM mitigated batch effects across all features, as indicated by comparing residual study effects to those in unharmonized data (Cohen's F‐squared: ComBatLS median = 0.0136, IQR = 0.032; ComBat‐GAM median = 0.0134, IQR = 0.033; Unharmonized median = 0.082, IQR = 0.100; Figure S29). However, there are differences in the centiles from data harmonized with ComBatLS or ComBat‐GAM (mean absolute difference in centile scores = 0.351, range = 0.039–5.05 centiles) with 42.2% of subjects having discrepant categorization of extremely high (< 5%) or low (> 95%) centiles in at least one feature (mean = 0.72 features per subject, range = 0–18 features). We conducted exploratory analyses to assess whether mean absolute differences in subjects' ComBatLS‐ and ComBat‐GAM‐harmonized centile scores varied as a function of the primary studies' characteristics, which may suggest that such differences are driven by ComBatLS's preservation of covariates like age and sex. This revealed subtle associations with the mean age of the study sample, the range of ages included in a sample, and how much an individuals' age deviated from that sample's mean age when controlling for the sample's size (Section S1A, Figure S30). The simulation studies described earlier (Figures 1, 2, 3, 4) suggest that ComBatLS's accurate preservation of scale effects, including age effects, contributes to the observed differences in centile scores from consortium data harmonized with ComBatLS compared to ComBat‐GAM. Finally, we demonstrated that ComBatLS's benefits are not dependent on the specific brain chart by fitting a different normative model based on prior literature, which produced consistent results (Section S1, Figure S31).

## 4 Discussion

Here we introduce and validate ComBatLS, a novel location‐ and scale‐preserving method to harmonize data across batches. Neuroimaging has begun to answer long‐standing questions about the brain's normative structure across the lifespan and to quantify individuals' deviation from this norm (Bethlehem et al. 2022; Dima et al. 2021; Frangou et al. 2022; Marquand et al. 2019). However, such studies require integrating vast quantities of MRI scans collected across studies, sites, and scanners, which are highly unlikely to have similar sample characteristics. While ComBat and its extensions preserve the effects of such biological covariates on features' means (J.‐P. Fortin et al. 2017; Pomponio et al. 2020), failure to preserve variance effects during harmonization may increase error and induce biases in downstream analyses. This includes normative scores derived from growth charts and estimates from brain‐age models, which are known to be impacted by harmonization (Lombardi et al. 2020; Yu et al. 2023). Furthermore, while additional work is needed to quantify ComBatLS's impact outside of normative modeling, which relies directly on features' scales, the relationship between variance and effect size implies that errors in scale could impact group‐level inferences (Kang et al. 2024). By integrating scale preservation in a robust batch‐correction framework and illustrating its ability to improve individuals' normative scores, this study and corresponding open software represent a significant advance in neuroimaging harmonization.

Leveraging structural features derived from nearly thirty thousand UK Biobank participants, we found that ComBatLS recapitulates subjects' true normative scores better than ComBat or ComBat‐GAM. We first used weighted sampling to create synthetic batches that were male‐dominated, female‐dominated, or balanced for sex. We then applied ComBatLS and several existing ComBat methods to “harmonize” these data and assessed how each method offset subjects' centile and Z‐scores from their true values. Given the random nature of these synthetic “batches,” we were able to effectively isolate the covariate‐preservation aspect of ComBatLS and quantify its effects independent of actual batch‐effect removal. Across 100 sampling replications and 208 brain features, we found that ComBatLS consistently produces normative scores closest to the ground truth, unharmonized data. This result remained stable across a range of sensitivity analyses, including removing subjects at the extreme ends of the normative distribution and when synthetic samples' sex differences were as small as 10%. These results indicate that by preserving the effects of biological covariates on distributions' scales, ComBatLS enables highly accurate quantifications of individuals' deviations from neuroanatomical norms.

ComBatLS's preservation of this critical intersubject variability is demonstrated by its performance in features with variances that are both strongly and minimally affected by sex. Across our analyses, harmonization methods were most comparable when applied to cortical thickness features, whose variances are less impacted by sex than other neuroanatomical phenotypes (Forde et al. 2020; Ritchie et al. 2018; Wierenga et al. 2018, 2022) (Section SIII). Yet even in cortical thickness, we found that ComBatLS still recapitulates each feature's true centiles better than the other methods in more than half of the replications. Thus, the relationship between ComBatLS's performance and the strength of sex effects on scale only emphasizes how important such effects are for centile estimation and should not discourage ComBatLS's use in features with minimal biological impacts on variance.

One motivation in developing ComBatLS was to prevent harmonization from inducing sex biases in normative scores by systematically inflating or deflating the centiles of either sex. Interestingly, our analyses reveal that none of the covariate‐preserving methods—ComBat, ComBat‐GAM, and ComBatLS—induce large differences in males' and females' centile scores (Section S1). However, ComBatLS induces less bias in centile errors across 100 replications, suggesting that covariate preservation in scale does mitigate harmonization‐induced biases.

We also validated that ComBatLS can harmonize neuroanatomical data across more than 50 real‐world studies with heterogeneous sample demographics, clearly demonstrating the practical scalability of ComBatLS to large studies. Exploratory assessment of differences in the centiles calculated from ComBatLS or ComBat‐GAM, which was developed to harmonize studies spanning wide age ranges (Pomponio et al. 2020), reveals that subjects' mean absolute differences increase with the mean age of their primary study's sample and when that mean age is further from their own. Given that primary study samples varied greatly in the ages—and ranges of ages—represented, we theorize that these differences result from ComBatLS's ability to preserve age's effects on variance that are confounded with technical batch effects and thus removed by ComBat‐GAM. While true centiles are unknown, these results alongside our simulation analyses suggest that by preserving covariate's impact on variance, ComBatLS may produce more accurate centile score estimates than ComBat‐GAM in real‐world datasets.

This study has several limitations. First, as with all ComBat methods, ComBatLS will only preserve covariates that have been pre‐specified. Given the substantial evidence for age and sex effects in variance, we suggest that ComBatLS users preserve these by default and use exploratory analyses to determine whether other covariates of interest impact measurements' scales. Here the model fit information provided by the package's plot.comfam() function may be particularly useful in determining whether covariates are adequately specified. Similarly, ComBat methods rely on some degree of between‐batch variation to accurately estimate covariate effects, as illustrated by the slight increase in absolute centile errors for all methods when our simulated sites were balanced for sex (see Figure 4B). Thus, while even small sampling imbalances seem sufficient for ComBatLS, researchers should take care when preserving covariates to avoid overfitting. Additionally, ComBatLS does not preserve covariate effects in or remove batch effects from higher‐order moments such as skew or kurtosis, which will necessitate new methods to resolve (Zhang et al. 2018). Finally, while popular, the existing cross‐sectional ComBat methods assessed here only represent one flavor of multi‐site harmonization (Bayer, Dinga et al. 2022; Bayer, Thompson et al. 2022; Gebre et al. 2023; Hu et al. 2023). It will be important for future work to compare ComBatLS to—or potentially integrate it with—other innovative and emerging frameworks (An et al. 2025; Bridgeford et al. 2024; de Boer et al. 2024; Li et al. 2021; Zhu et al. 2024). Specifically, multivariate methods such as CovBat (Chen et al. 2022), DeepComBat (Hu et al. 2024), and DeepResBat (An et al. 2025), while useful in preserving features' complex relationships with other features and covariates, retain ComBat's basic assumptions in that covariates' effects on scale are not specifically preserved. As An et al. demonstrate, deep learning harmonization methods are not impervious to confounding batch and covariate effects, motivating the authors to argue for the explicit ComBat‐inspired preservation of covariates. Our results clearly support extending this logic to preserving covariate effects in features' scales; thus, future iterations may benefit from merging frameworks to apply ComBatLS in tandem with covariance harmonization (CovBat), in features' latent space (DeepComBat), or implemented via machine learning (DeepResBat).

The current study introduces ComBatLS, an R‐based harmonization tool that extends the ComBat framework by preserving the effects of biological covariates on both location and scale. Its ability to robustly estimate individuals' normative scores may enable sensitive identification of disease effects, increasing the power of future clinical research (Bethlehem et al. 2022). Furthermore, widespread sex differences in the variances of measures beyond neuroanatomy (Beery 2018; Khodursky et al. 2022; Zajitschek et al. 2020) indicate that ComBatLS may be useful for correcting batch effects across a broad range of disciplines. ComBatLS is openly available at https://github.com/andy1764/ComBatFamily.

## Author Contributions

Conceptualization: M.G., R.T.S., A.A.C., and A.F.A.‐B. Methodology: M.G., R.T.S., A.A.C., and A.F.A.‐B. Software: A.A.C. Formal analyses: M.G. Resources: J.S., R.A.I.B., and L.B.C.C. Data curation: R.A.I.B., R.R.‐G., V.W., and J.S. Writing – original draft: M.G. Writing – review and editing: M.G., R.T.S., R.A.I.B., R.R.‐G., V.W., L.D., S.S., P.T., J.S., A.F.A.‐B., A.A.C. Visualization: M.G. Supervision: A.F.A.‐B., A.A.C.

## Ethics Statement

The research was reviewed by The Children's Hospital of Philadelphia's Institutional Review Board (IRB 20‐017874) and deemed not to require PRE or IRB oversight as it consists of secondary analysis of de‐identified primary datasets. Informed consent of participants (or their guardians) in primary studies is available in Bethlehem et al. (2022).

## Conflicts of Interest

J.S., R.A.I.B., and A.F.A.‐B. hold shares in and J.S. and R.A.I.B. are directors of Centile Biosciences Inc. R.T.S. has received consulting income from Octave Bioscience and compensation for scientific reviewing from the American Medical Association. A.A.C. receives compensation for reviewership duties from the American Medical Association.

## Supporting information


Data S1.

## Data Availability

No new data were created or analyzed in this study. Data from the UK Biobank are available at https://www.ukbiobank.ac.uk/. Data included in the Lifespan Brain Chart Consortium is described in (Bethlehem et al. 2022). Links to open datasets are also listed at https://github.com/brainchart/Lifespan. All code used to perform analyses and create figures is available at https://github.com/BGDlab/combat‐biovar. ComBatLS can be found at https://github.com/andy1764/ComBatFamily.
