# Supplementary material for: ComBatLS: A Location‐ and Scale‐Preserving Method for Multi‐Site Image Harmonization
Source: Hum Brain Mapp. 2025 Jun 11;46(8):e70197. doi: 10.1002/hbm.70197 (PMC12152769; doi:10.1002/hbm.70197)
Supplement: Supplementary file 1 — Data S1. [file HBM-46-e70197-s001.pdf]

Supplementary Material

Contents

I. Demographic imbalances are common across sites in consortia and multisite samples
II. Results including ComBat without covariate preservation
III. The magnitude of sex's effect on variance depends on the type of brain feature measured
IV. Replication without Extreme Centiles
V. Z-score analyses
  A. Main Analyses
  B. Varying M:F ratios
  C. Replication without Extreme Z-scores
VI. Comparisons of ComBatLS and ComBat-GAM for harmonizing consortium data
  A. Main Analyses
  B. Validating generalizability to other normative models
VII. References

Section I. Demographic imbalances are common across sites in consortia and multisite samples

Like all ComBat methods, ComBatLS is designed to retain biological information that may be confounded by site effects. While some multi-site studies ensure that demographics are relatively uniform across sites, substantial variability in subject-level factors like sex and age are common (SFigs 1 and 2). These imbalances become even more prevalent when compiling datasets to create large, representative consortium samples, as is often done for normative modeling (SFig 3). Thus, is it cruical that harmonization is able to disambiguate and preserve covariate effects from nuisance site effects, both in brain feature distributions' locations (all ComBat methods) and scales (ComBatLS).

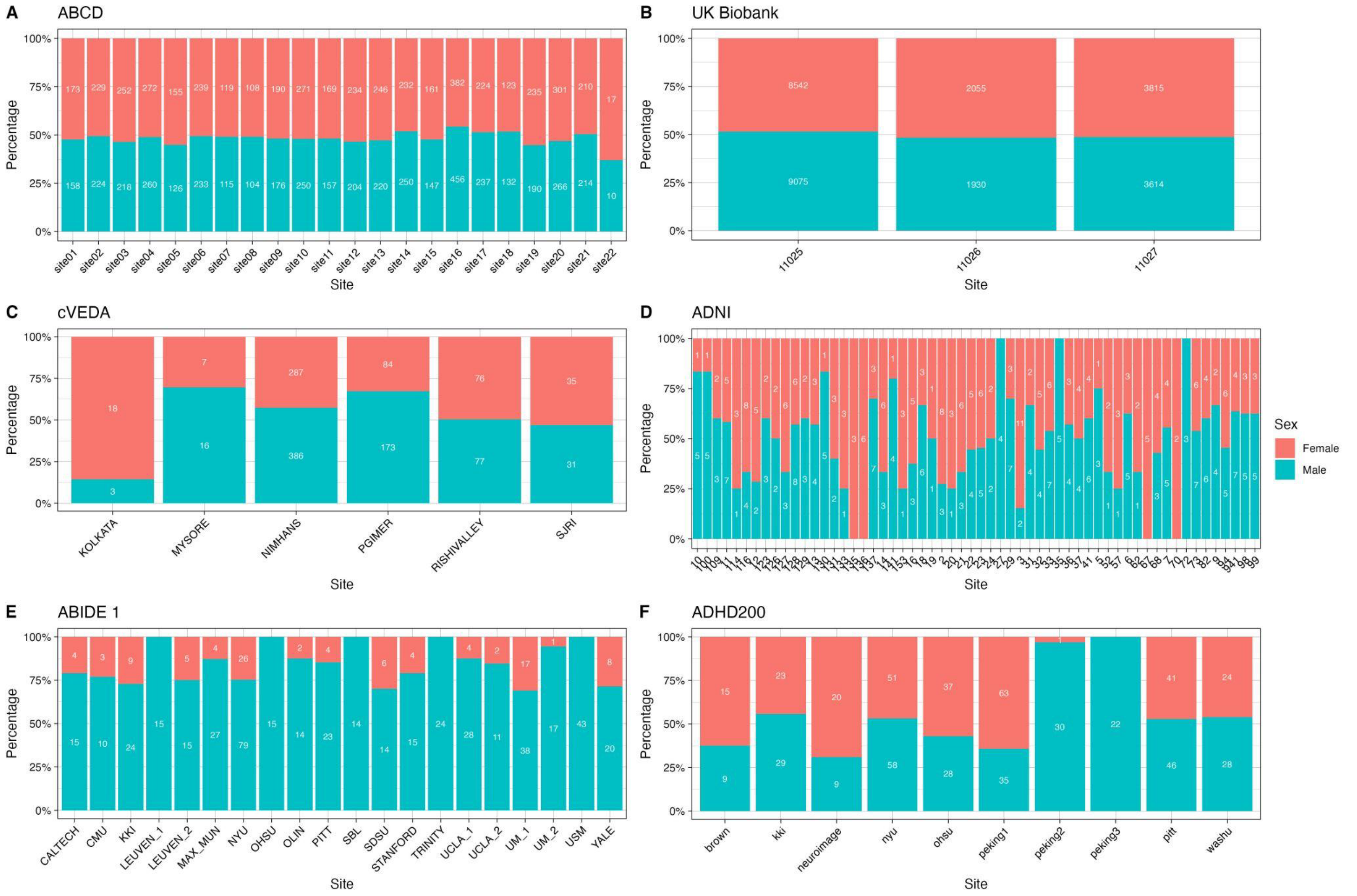

Supplemental Figure 1) In many multisite studies, the sex-ratios of each site's sample varies considerably. Bar plots indicate the percentage of each site's sample reported as Female or Male across six multisite studies included in the LBCC. Though the sex-ratios remain fairly stable in (A) the Adolescent Brain Cognitive Development (ABCD) and (B) UK Biobank, (C-F) the remaining studies's sex-ratios vary substantially across sites. Bars are labeled to show the count of males and females, respectively, in each site.

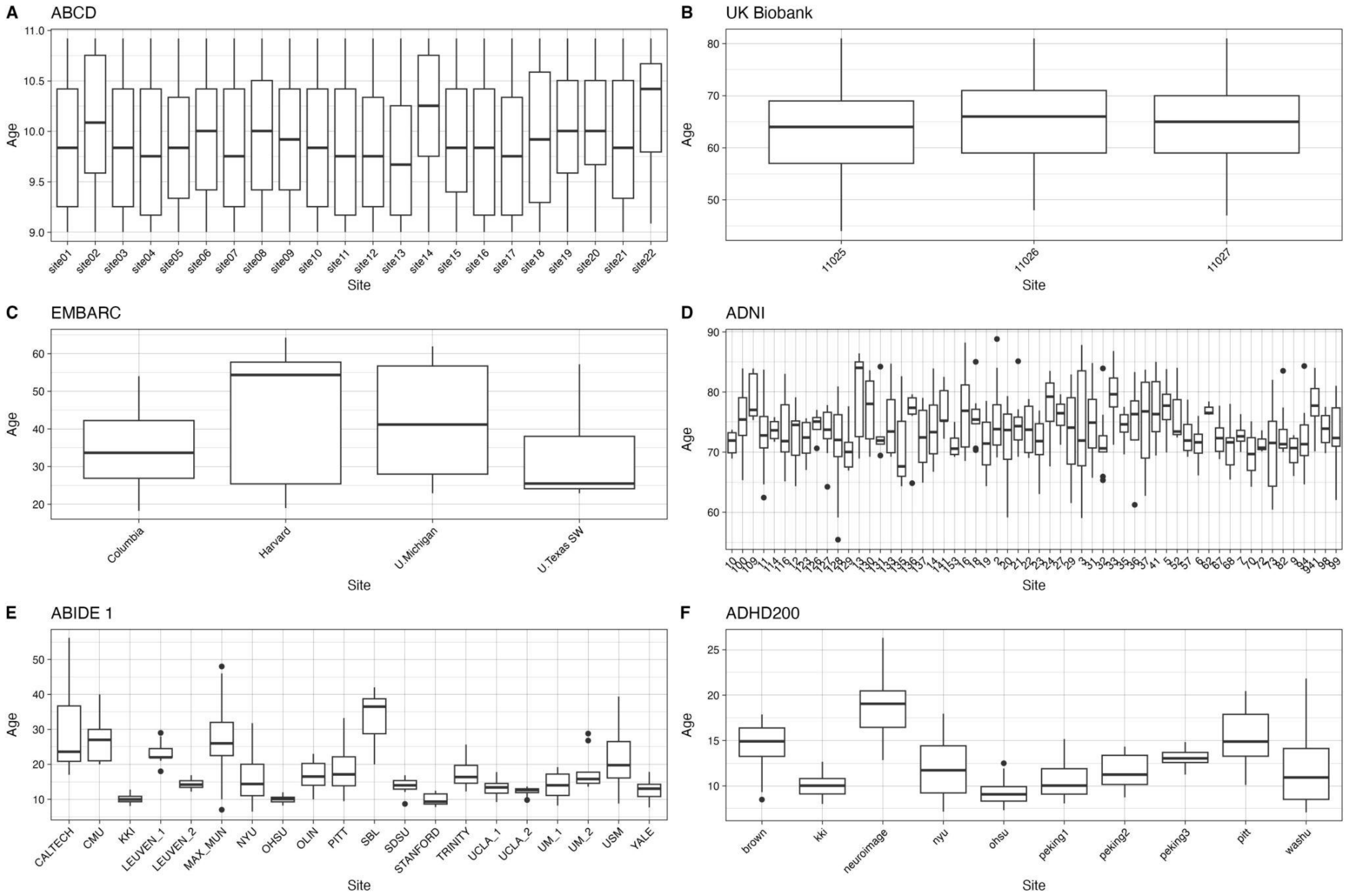

Supplemental Figure 2) Sample's ages are often inconsistent across studies' sites. Box and whisker plots indicating the age (years post-birth) of each site's sample across six multisite studies in the LBCC. (A and B) While some studies' sites have very similar age distributions, (C-F) the subjects' age distributions are often vary greatly from one site to another.

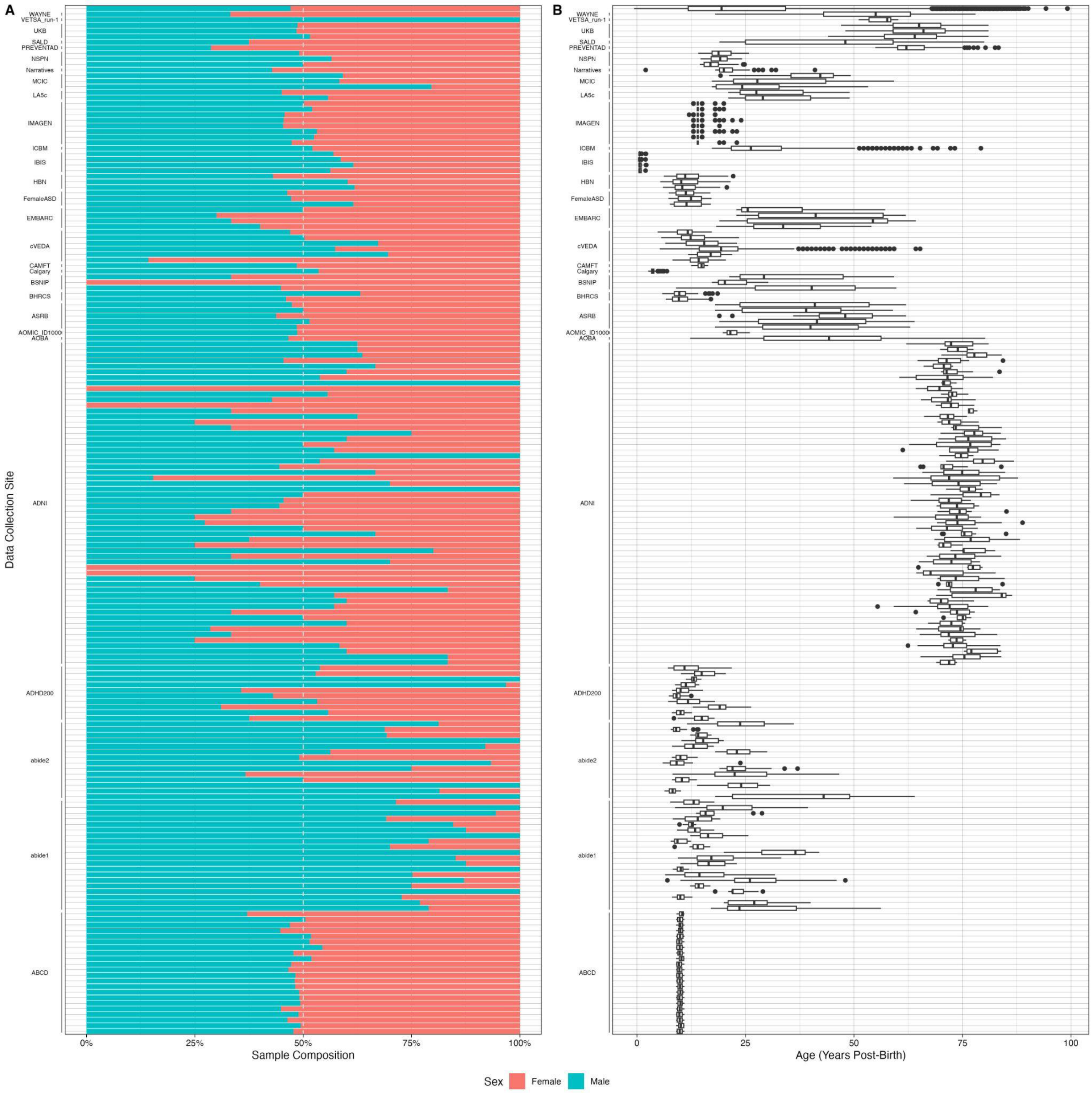

Supplemental Figure 3) Consortium samples are particularly likely to show site-level variability in sample demographics. A) Sample sex-ratios by site and study. B) Age distribution of each site's sample, grouped by study.

Section II. Results across 100 sampling replications including ComBat without Covariate Preservation

We included an application of ComBat in which no covariate effects are preserved to serve as a benchmark harmonization method against which we could assess the effects of increasingly complex covariate preservation: linear ComBat, ComBat-GAM, and ComBatLS. While tests with this method are controlled for throughout the analyses as part of our FDR corrections, we chose not to include results in the main text to

facilitate easy comparison of our primary methods of interest. Results from all four methods are presented below.

Pairwise comparisons of ComBat methods' absolute centile errors for 208 brain features across 100 replications		
**Method Producing Smaller Absolute Errors**	**N Features**	**% Features**
ComBat-GAM vs ComBat w/o Covariates		
ComBat w/o Covariates	190	0.91%
ComBat-GAM	20605	99.06%
ComBatLS vs ComBat w/o Covariates		
ComBat w/o Covariates	155	0.75%
ComBatLS	20638	99.22%
ComBatLS vs ComBat-GAM		
ComBat-GAM	1503	7.23%
ComBatLS	19115	91.90%
Linear ComBat vs ComBat w/o Covariates		
ComBat w/o Covariates	190	0.91%
Linear ComBat	20604	99.06%
Linear ComBat vs ComBat-GAM		
ComBat-GAM	8093	38.91%
Linear ComBat	9918	47.68%
Linear ComBat vs ComBatLS		
ComBatLS	19146	92.05%
Linear ComBat	1466	7.05%

Supplemental Table 1) Pairwise tests of absolute centile errors within each of 208 brain features replicated across 100 subject resamplings. All tests were conducted as pairwise, two-tailed t-tests of ranks with Welch's correction. FDR-corrected across 1248 tests (208 features x 6 ComBat method pairings) within each sampling permutation.

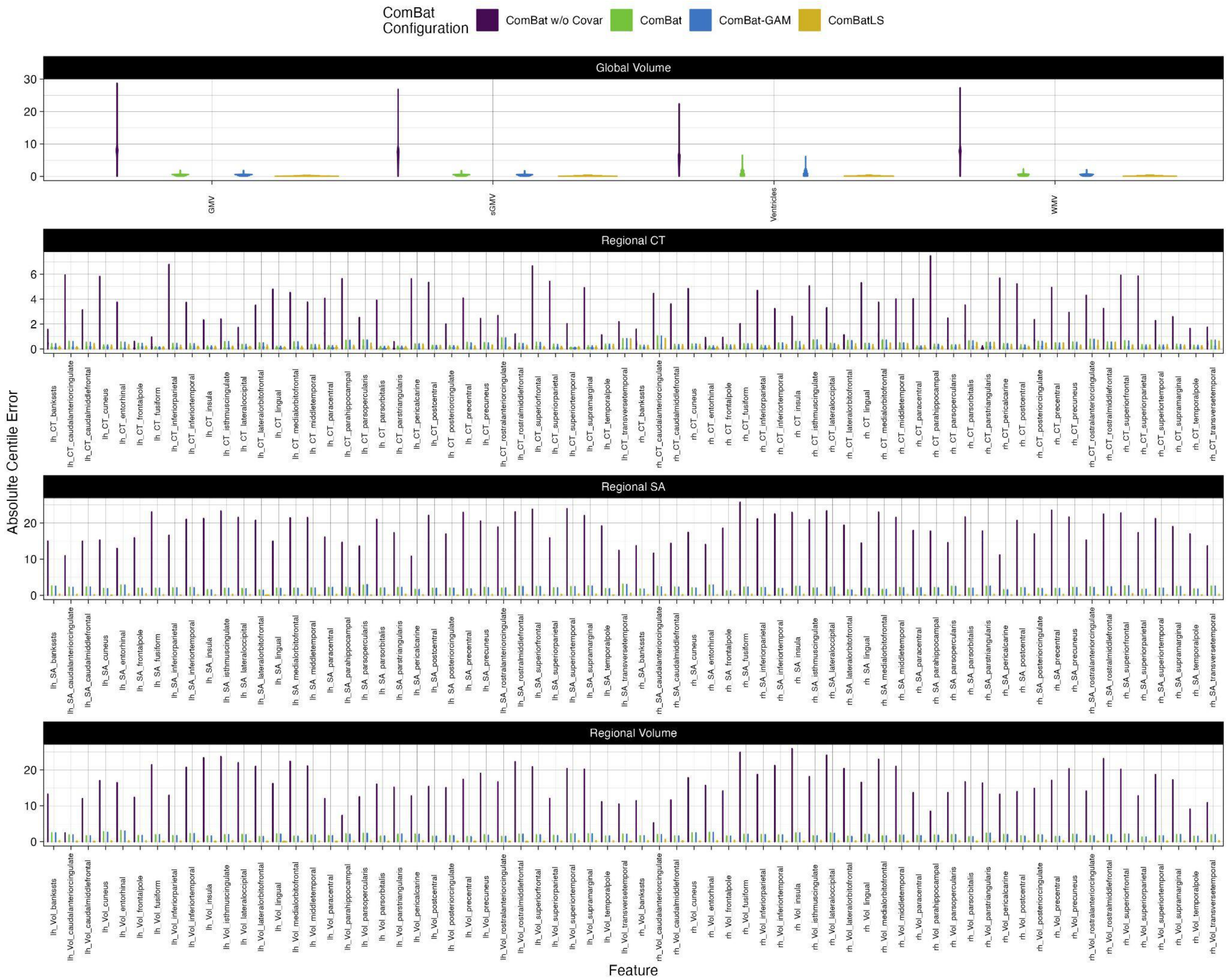

Supplemental Figure 4. Absolute centile errors across brain features and ComBat methods. Violin plots of absolute centile errors across 208 brain features.

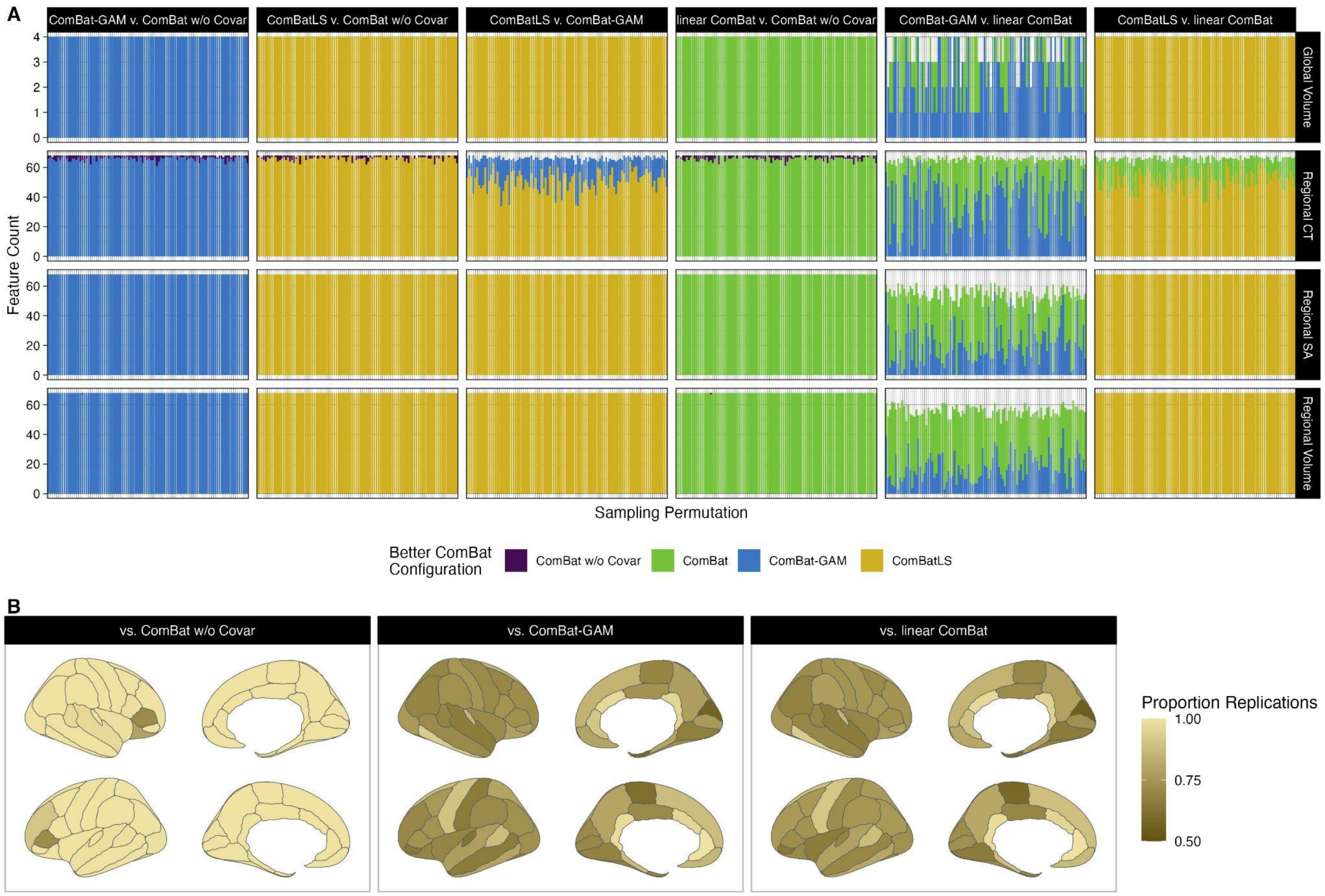

Supplemental Figure 5. ComBatLS recapitulates true centile scores more accurately than other ComBat methods. A) Absolute centile errors within each brain feature compared pairwise between four ComBat methods, replicated across 100 sampling permutations. Fill indicates the ComBat method that produces significantly smaller absolute centile errors, FDR-corrected across 1248 tests (208 features x 6 ComBat method pairings) within each permutation. B) Proportion of sampling replications in which ComBatLS produces significantly smaller absolute centile errors for a cortical thickness feature than an alternative ComBat method. Significant differences were assessed using pairwise t-tests between ComBat methods, FDR-corrected. Abbrv: CT, cortical thickness; SA, surface area.

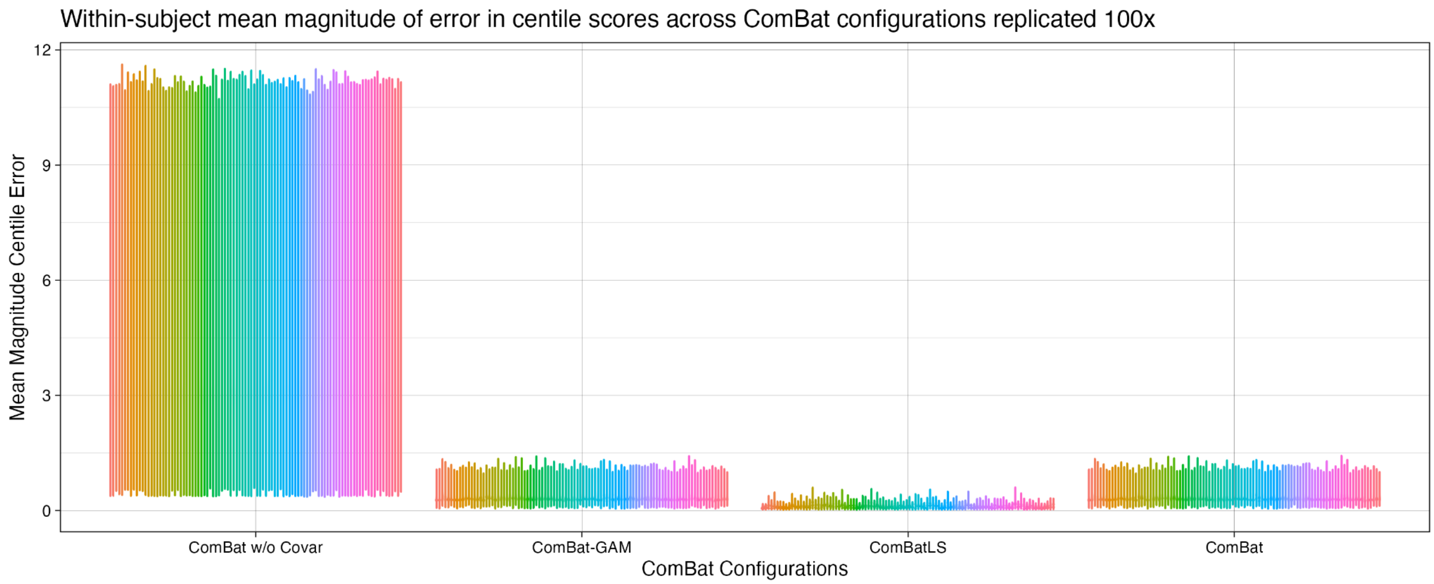

Supplemental Figure 6. Subjects' mean absolute centile error across ComBat configurations and 100 sampling replications. Violin plots of absolute centile error for 208 features averaged within subject. Fill corresponds to sampling replication.

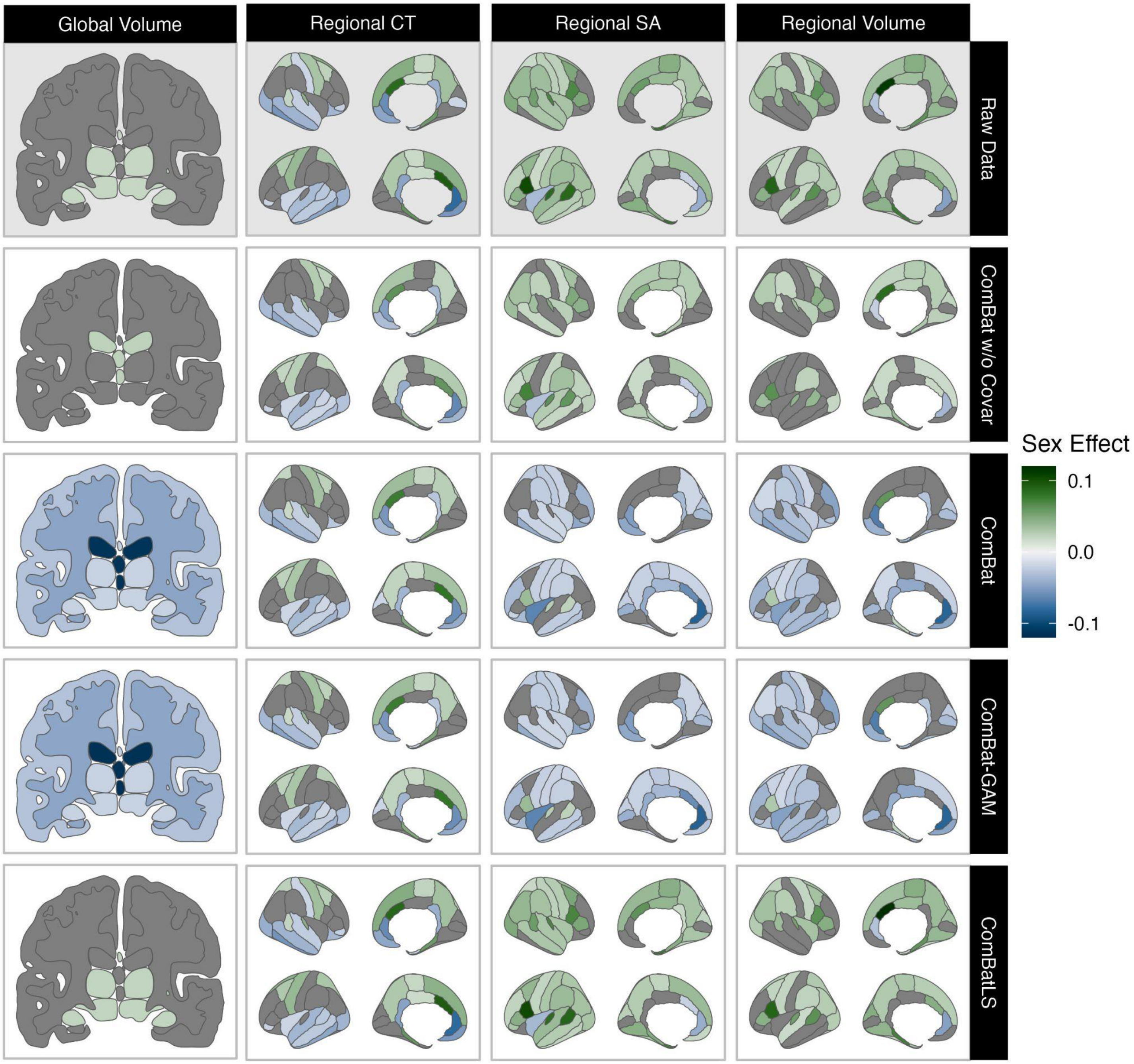

Supplemental Figure 7. Brain features with significant sex effects in scale. Features in gray are those for which sex does not significantly impact the second moment of a gamlss brain chart. Fill represents the difference in males' and females' predicted variance at the sample's mean age (64.94 years), standardized by dividing by females' predicted variance. Positive effects indicating that males' variance is higher than females'.

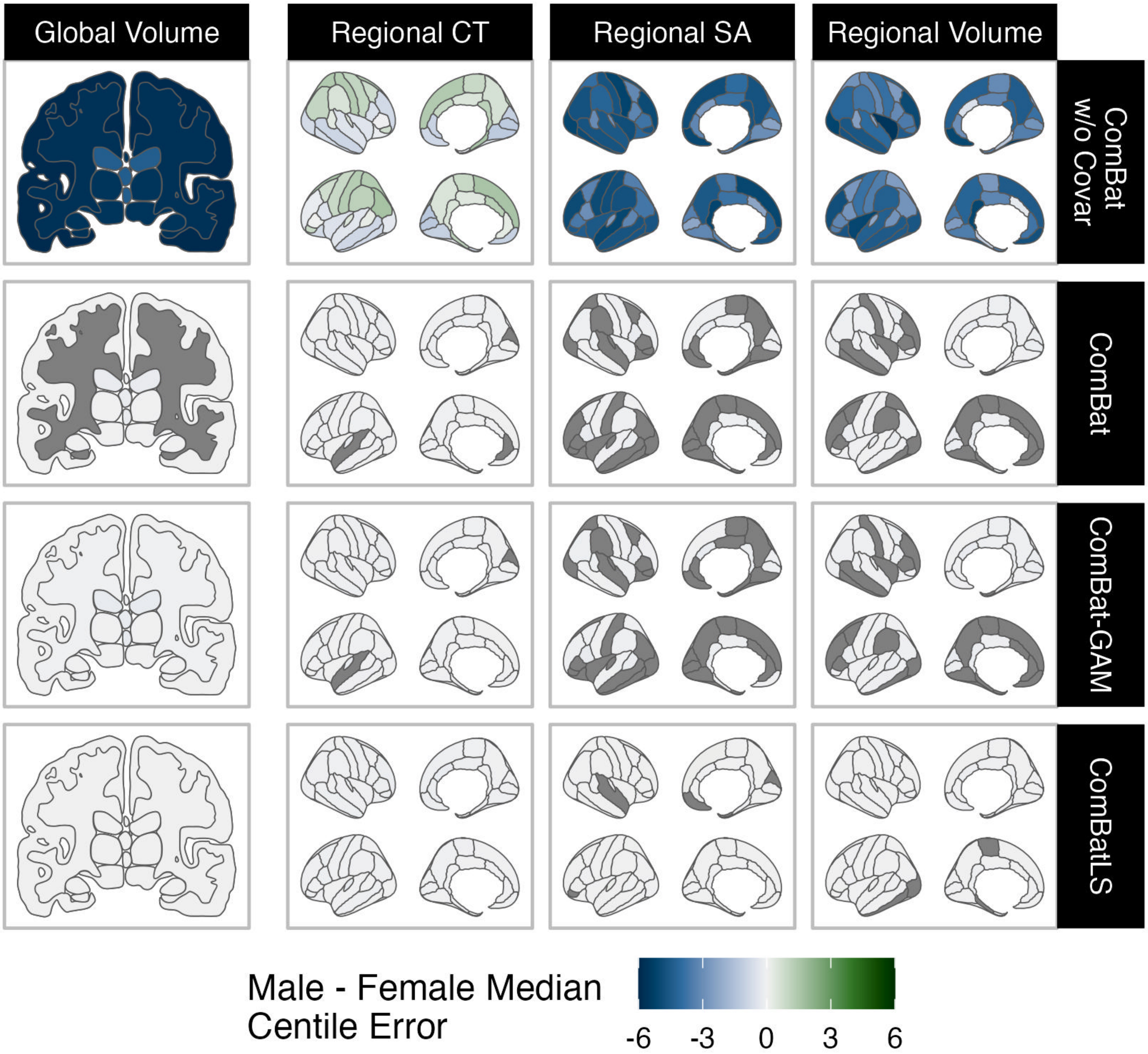

Supplemental Figure 8. Significant differences in males' and females' median centile errors across brain features and ComBat methods. Positive centile errors (green) indicate that males' centiles tend to be overestimated relative to females'. Abbrv: CT, cortical thickness; SA, surface area. Results did not change when correcting for multiple comparisons.

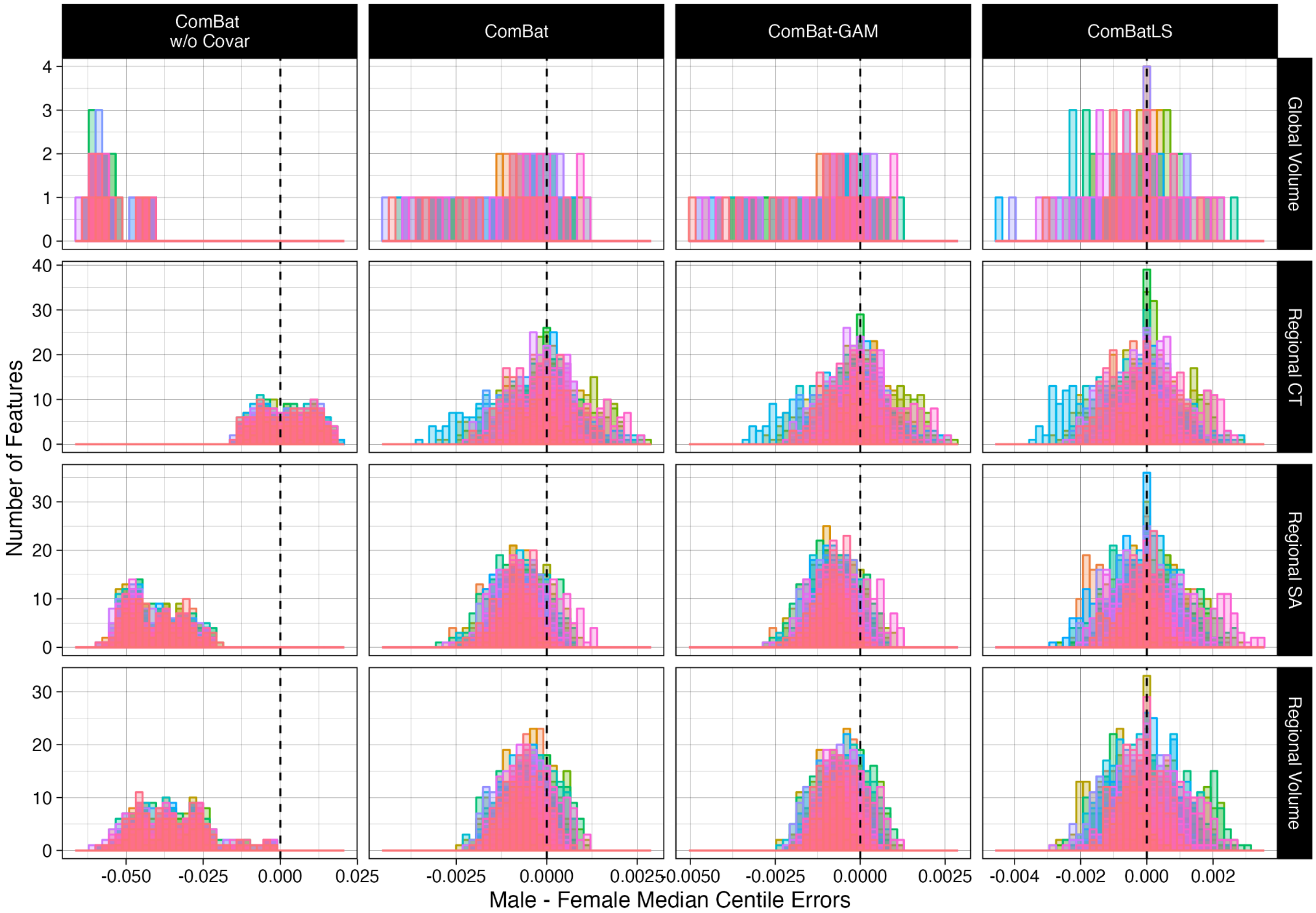

Supplemental Figure 9. Differences between males' and females' median centile errors in 208 brain features across 100 sampling replications. Feature counts are plotted by brain tissue types. Fill represents sampling replication. Abbrv: CT, cortical thickness; SA, surface area.

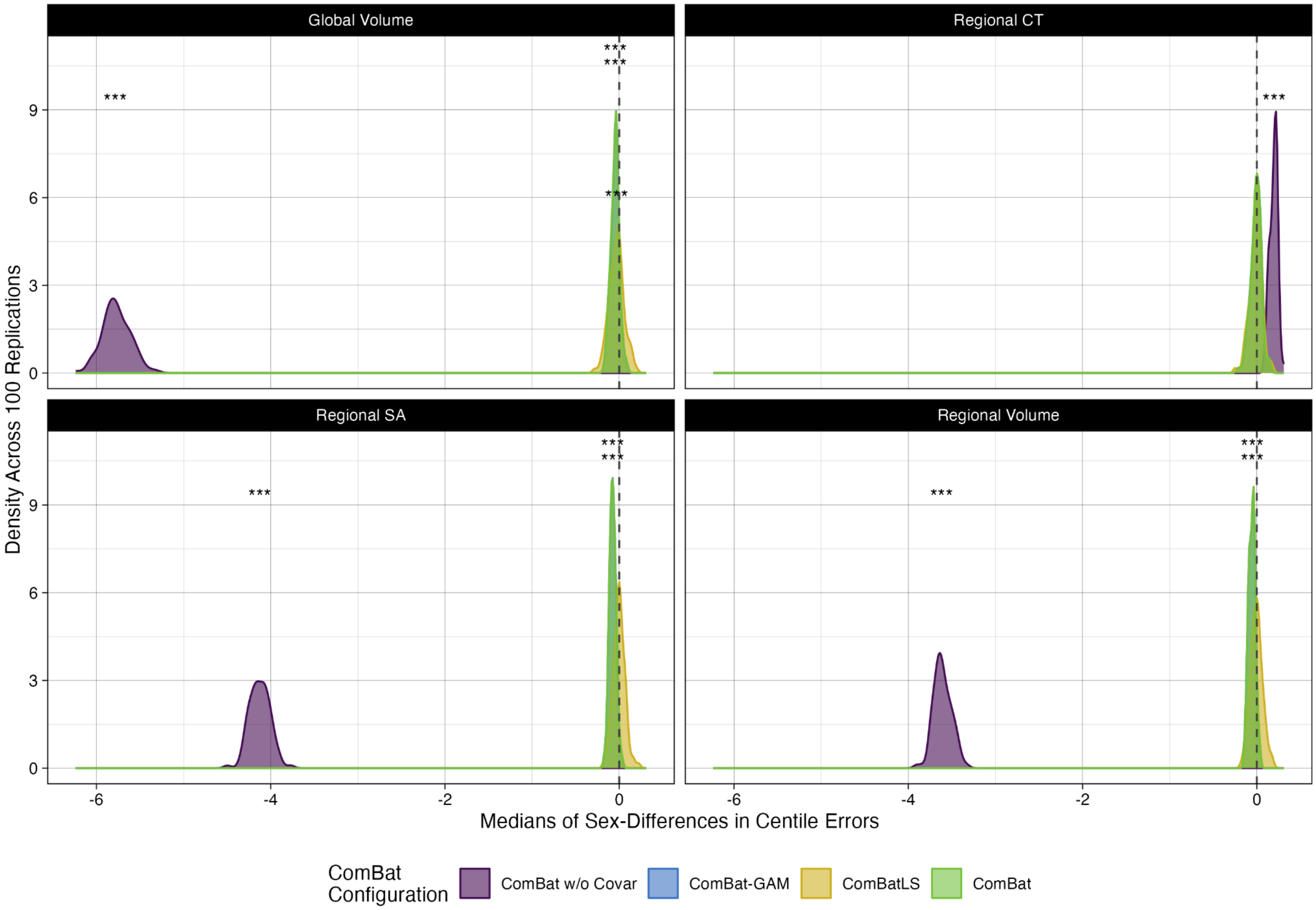

Supplemental Figure 10. Density plots of median sex differences in centile errors induced by different ComBat methods within phenotype categories across 100 replications. Data shows medians of the distributions of each replication plotted in Supplemental Figure 5. Abbrv: CT, cortical thickness; SA, surface area; ***, $p < 0.001$; **, $p < 0.01$. Results did not change when correcting for multiple comparisons.

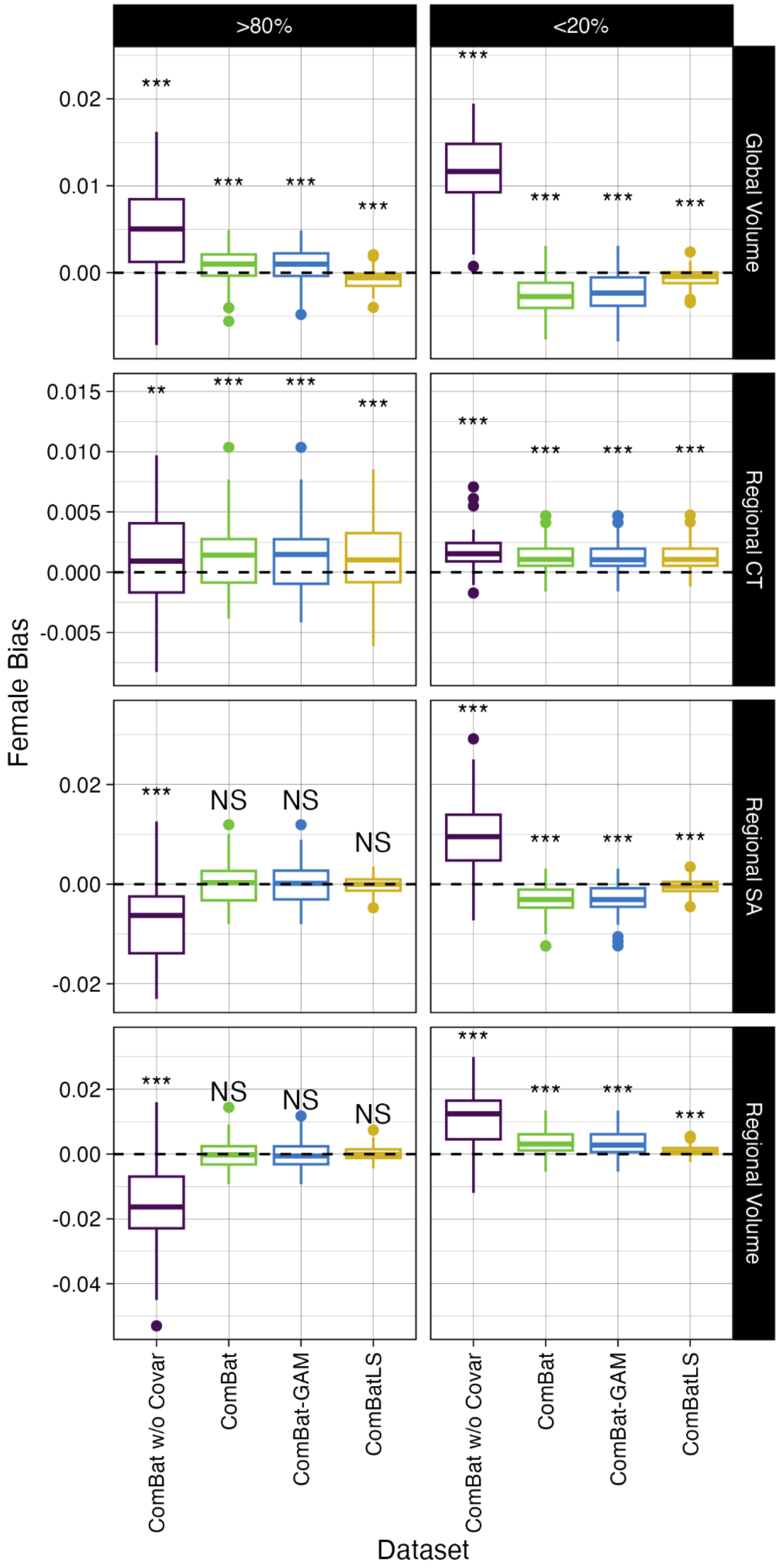

Supplemental Figure 11. ComBat methods may lead females to be slightly over- or under-represented among individuals with extreme phenotypes. Bias in the proportion of females with low (<20th percentile) or high (>80th) mean centiles across 100 sampling replications. Positive values indicate a higher proportion of females than “true” mean centiles calculated from unharmonized data (dashed line). Abbrv: ***, p < 0.001; **, p < 0.01.

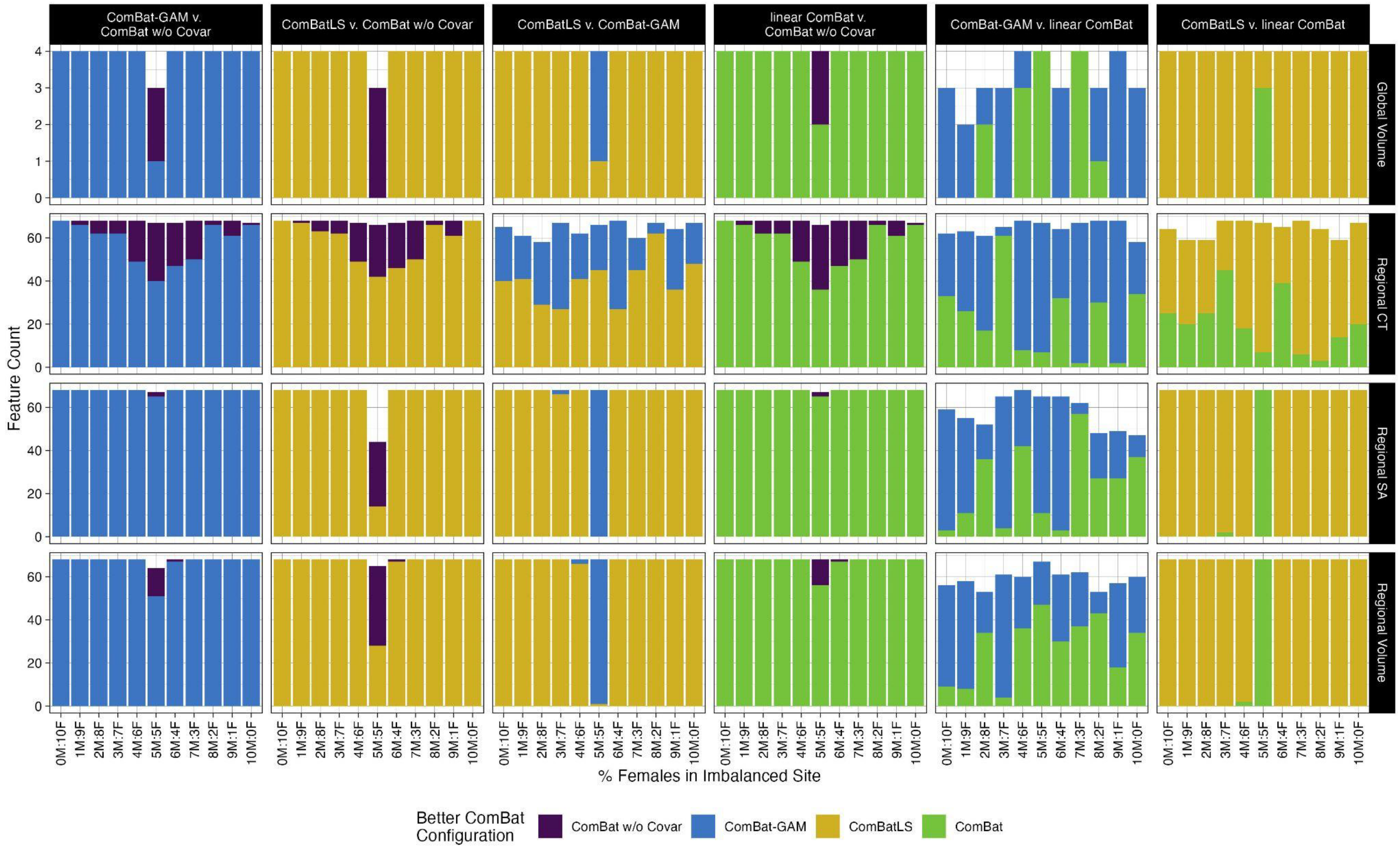

Supplemental Figure 12. Magnitude centile errors compared pairwise between ComBat methods across varying levels of sex-imbalances in simulated sites. Fill indicates ComBat method with significantly lower absolute centile errors for a given feature, FDR-corrected across pairwise combinations, brain features, and 11 samplings. Abbrv: CT, cortical thickness; SA, surface area.

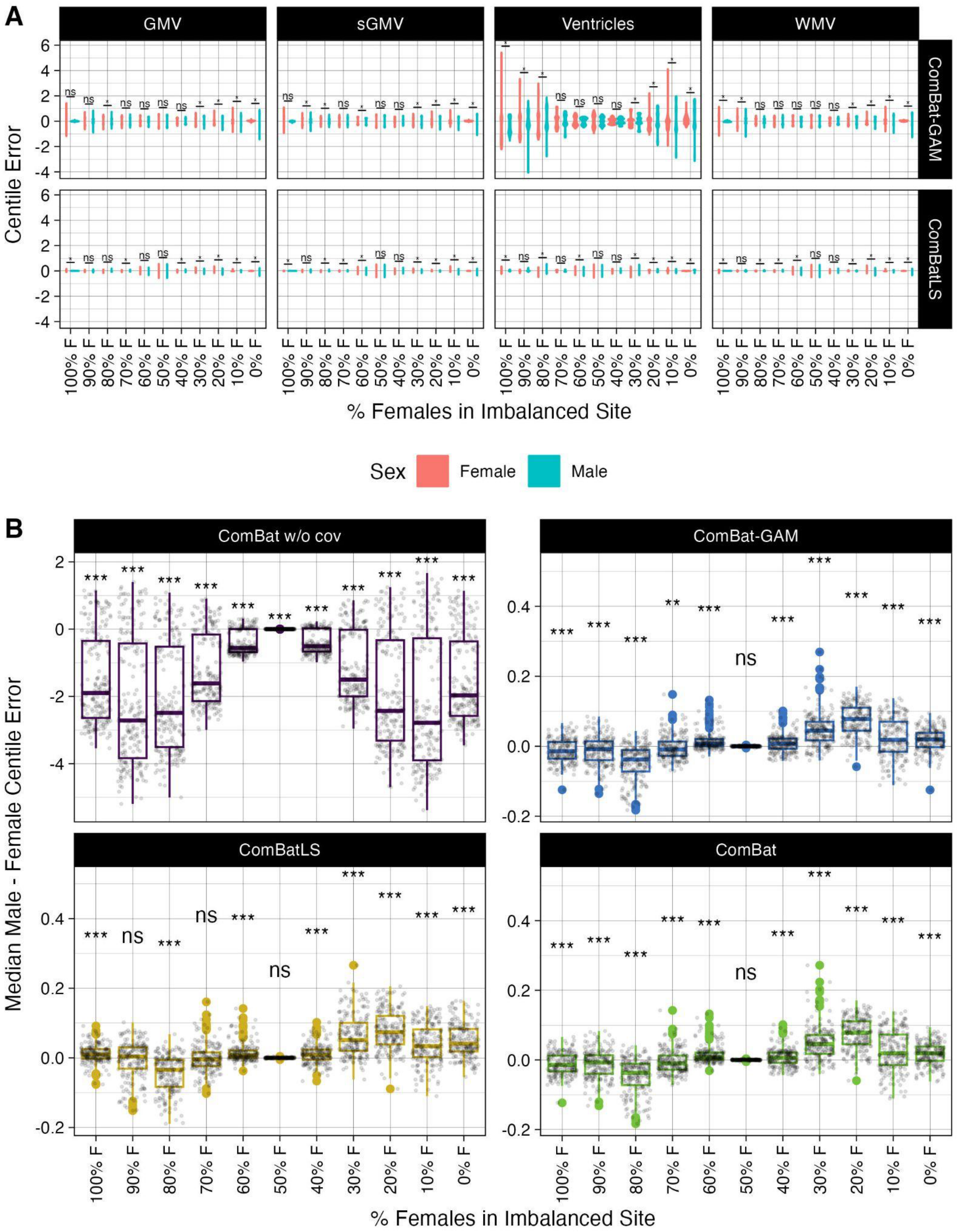
A
GMV
sGMV
Ventricles
WMV
ComBat-GAM
ComBatLS
Centile Error
% Females in Imbalanced Site
Sex
Female
Male
B
ComBat w/o cov
ComBat-GAM
ComBatLS
ComBat
Median Male - Female Centile Error
% Females in Imbalanced Site
100% F
90% F
80% F
70% F
60% F
50% F
40% F
30% F
20% F
10% F
0% F

110

Supplemental Figure 13. Sex-biases in centile errors induced by various ComBat methods across varying degrees of sex-imbalance. A) Illustrative example of differences in males' and females' distributions of centile errors across global brain features when harmonized with ComBat-GAM and ComBatLS. B) Points show differences in males' and females' median centile errors in each brain feature. Boxplots show median male - median female centile errors across these features when centiles are derived from data harmonized by different ComBat methods. ComBat without covariate preservation induces strong biases wherein males' centiles are underestimated relative to females', particularly as simulated sites become more imbalanced for sex. Results for both panels are highly similar when applying FDR correction across 11 M:F ratios. Abbrv: ***, $p < 0.001$; **, $p < 0.01$.

Section III. The magnitude of sex's effect on variance depends on the type of brain feature measured

Motivated by prior literature, we used UKB data to assess how sex's effects on scale vary by brain feature type. Sex's effects in variance were calculated from brain charts and standardized across features (see Methods). We performed a nonparametric Kruskal-Wallis test to establish that the distributions of standardized sex effects varied across brain feature types: global tissue volumes (4 features), regional cortical thickness (68 features), regional surface area (68 features), and regional volume (68 features). We then conducted pairwise Wilcox tests (FDR-corrected) which show that cortical thickness features's scales are significantly less impacted by sex than cortical surface area or cortical regional volume features. Sex-effects on cortical thickness features' scales are not significantly smaller than those of global volumes, though this analysis is limited by small number of global volume features.

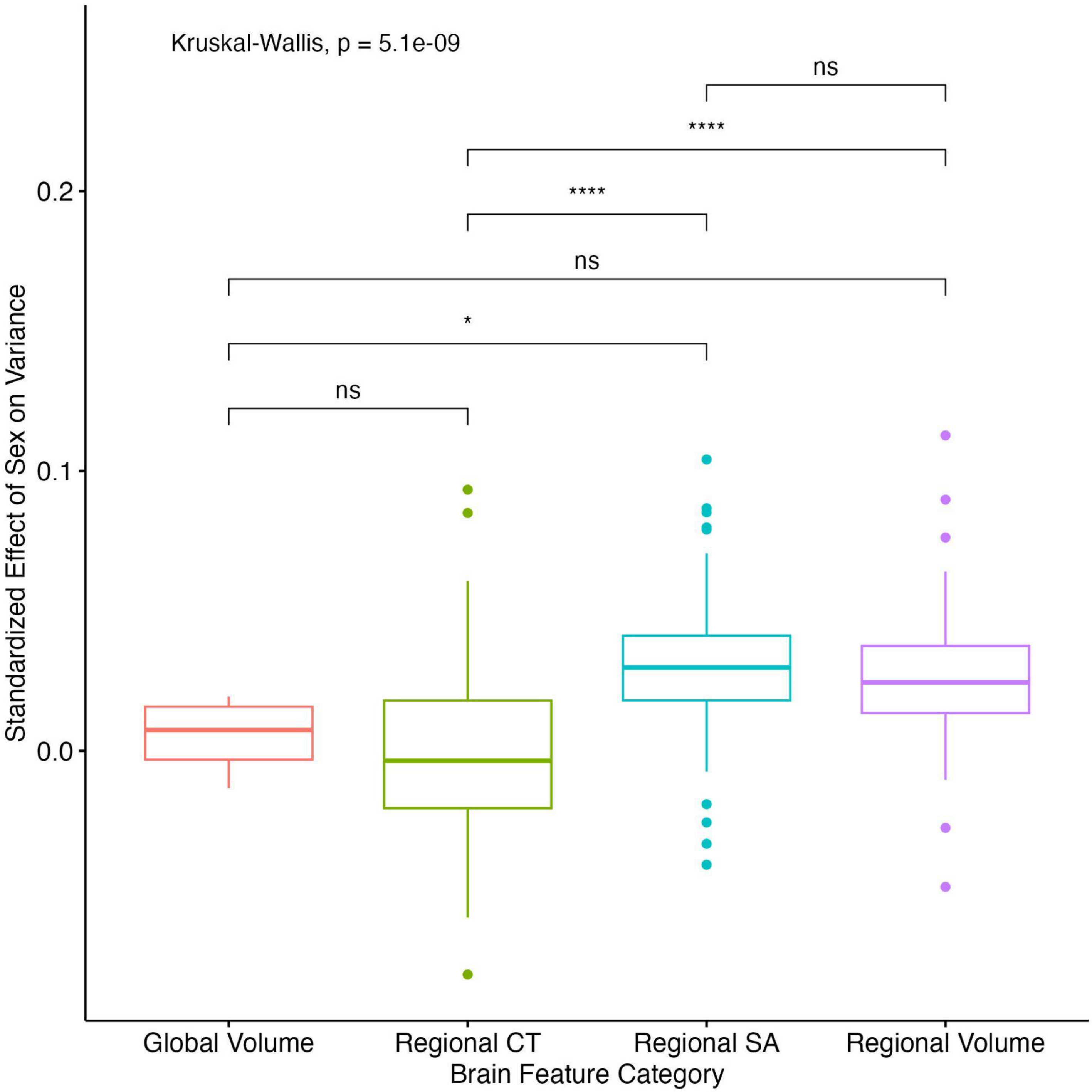

Supplemental Figure 14. Sex has smaller effects on cortical thickness features' variances than other regional phenotypes. Abbrv: CT, cortical thickness; SA, surface area; ***, p < 0.001; **, p < 0.01, FDR-corrected.

Section IV. Replication without Extreme Centiles

To determine whether a small number of subjects with very high or very low centile scores drove differences across ComBat methods, we repeated our statistical comparisons after removing subjects with “extreme” centiles in a given feature. We defined extreme centiles as those >95% or <5% when calculated from raw, “unharmonized” data. First, we identified and removed extreme centiles across our 100 replications. We then compared the remaining centile errors across ComBat methods, using two-tailed t-tests of centile error ranks with Welch’s correction, controlling FDR for 1248 comparisons within each replication. We also repeated our assessments of sex-differences in centile errors and ComBat-induced sex biases in centile displacement after taking the median of sex-differences within feature categories. Second, we applied these procedures to our 11 samples of synthetic sites with varying Male:Female ratios to assess whether extreme subjects drove differences in ComBat methods’ performance across degrees of sex imbalance.

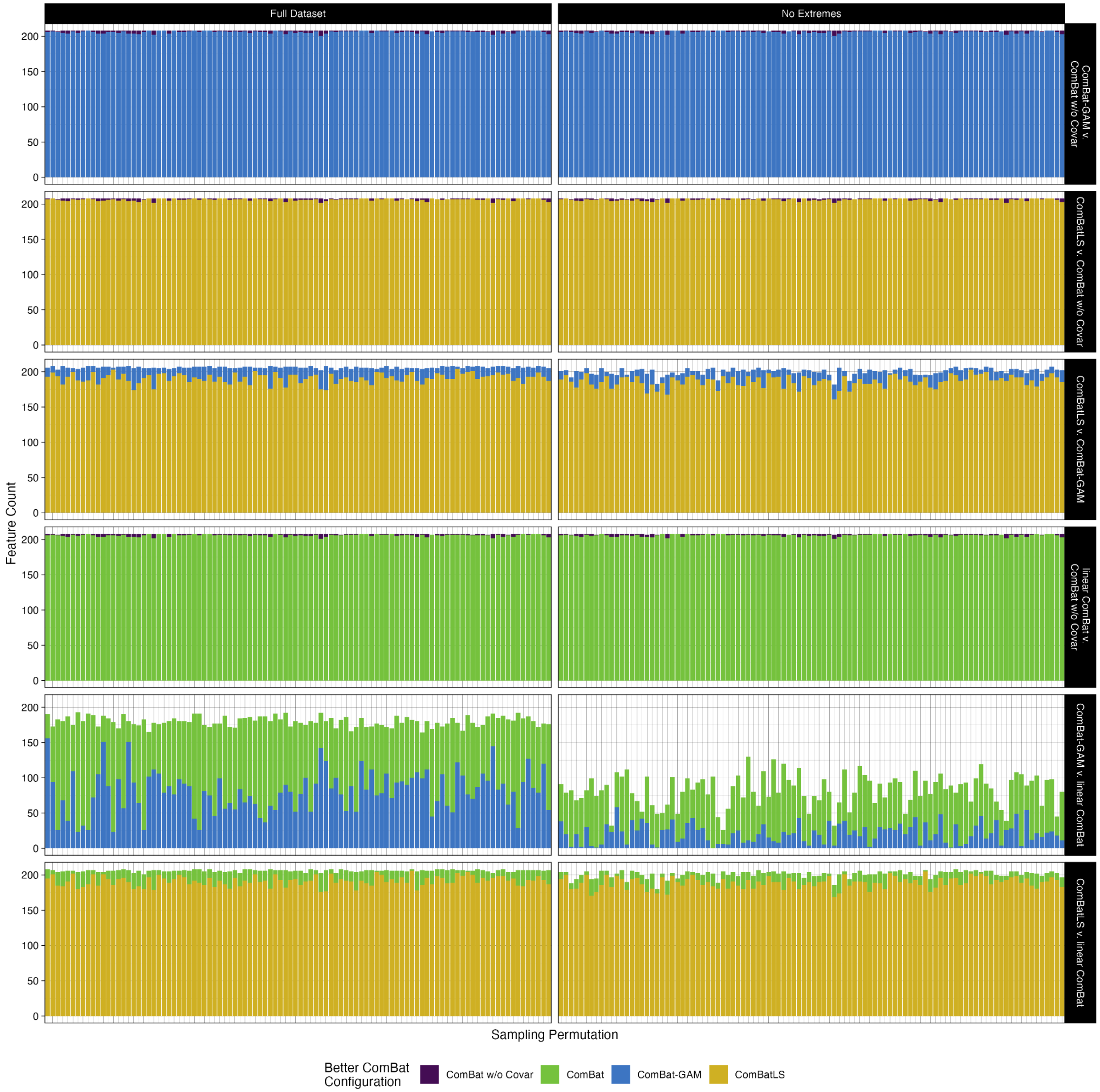

Supplemental Figure 15. Extreme phenotypes do not drive differences in absolute centile errors between ComBat methods. Comparison of pairwise tests of absolute centile errors between ComBat methods when centiles with raw values above 95% or below 5% are excluded. Absolute centile errors were compared within each brain featureacross 100 sampling permutations. Fill indicates the ComBat method that produces significantly smaller absolute centile errors, FDR-corrected across 1248 tests (208 features x 6 ComBat method pairings) within each permutation.

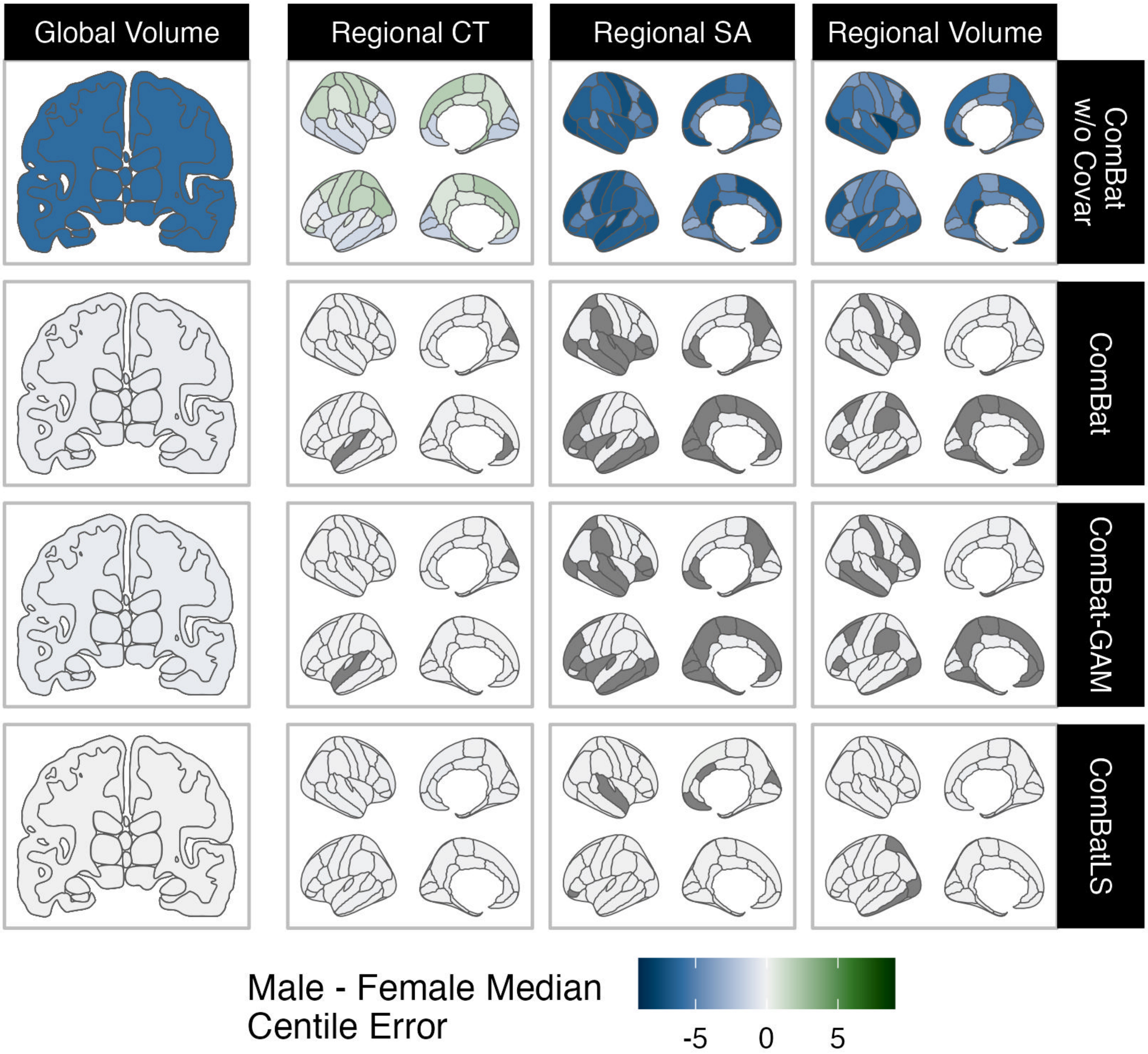

Supplemental Figure 16. Significant differences in males' and females' median centile errors across brain features and ComBat methods when extreme features are excluded. Positive centile errors (green) indicate that males' centiles tend to be overestimated relative to females'. Results do not change when correcting for multiple comparisons. Abbrv: CT, cortical thickness; SA, surface area.

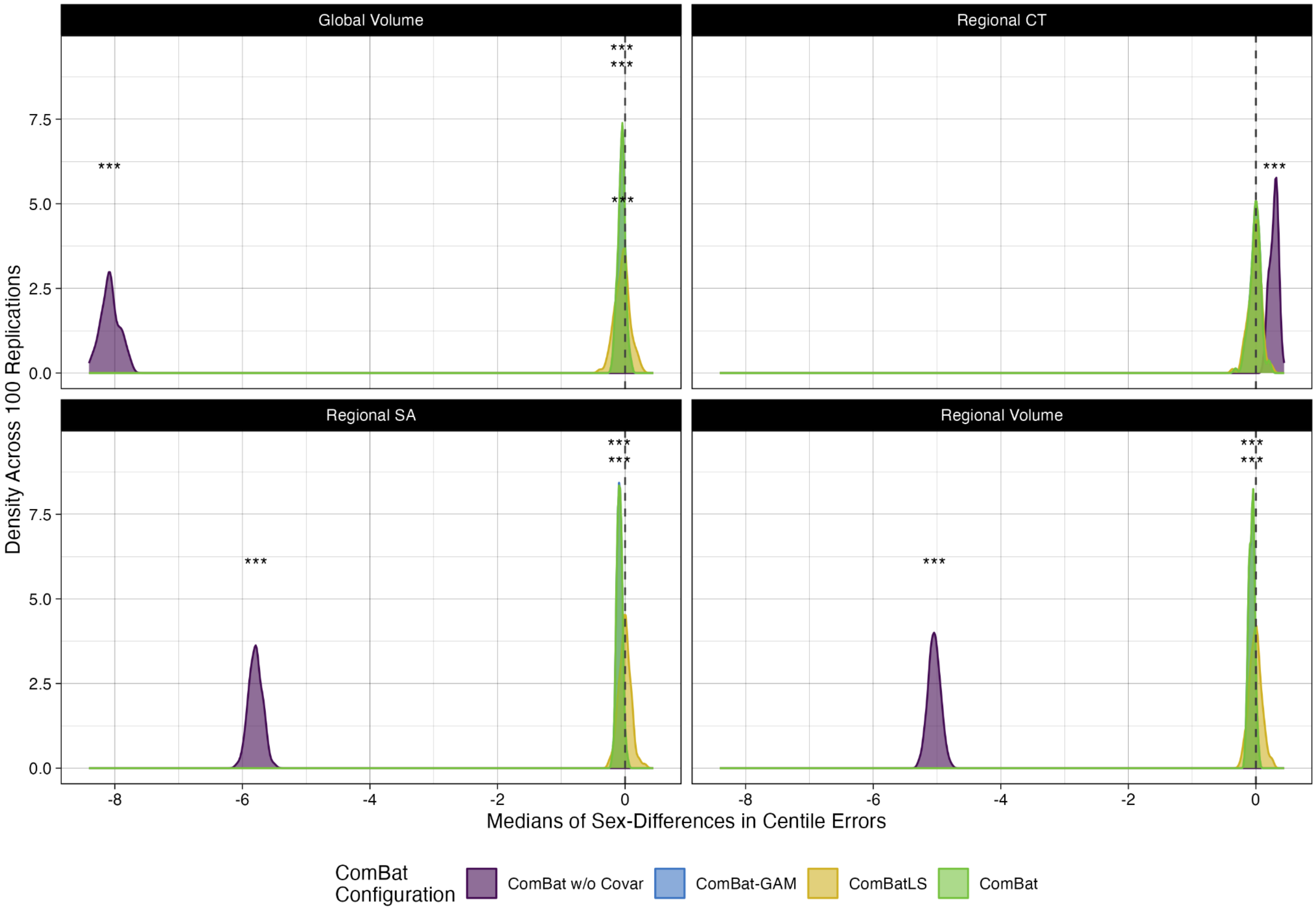

Supplemental Figure 17. Density plots of median sex differences in centile errors induced by different ComBat methods across 100 replications when extreme phenotypes are excluded. Results are consistent when applying FDR-correction across 4 phenotype classes and 4 harmonization methods. Abbrv: CT, cortical thickness; SA, surface area; ***, $p < 0.001$; **, $p < 0.01$.

Section V. Z-score analyses

Z-scores for each feature derived from centile scores using R's *qnorm()* function. To prevent infinite z-scores, centiles of 0 and 1 were estimated as 1e-25 and 0.99999999999999994, respectively. As with centile scores, analyses of z-scores were repeated without extreme scores, here defined as z-scores less than -2 or greater than 2.

A) Main Results

Pairwise comparisons of ComBat methods' absolute Z-score errors for 208 brain features across 100 replications

Method Producing Smaller Absolute Errors	N Features	% Features
ComBat-GAM vs ComBat w/o Covariates		
ComBat w/o Covariates	191	0.92%
ComBat-GAM	20600	99.04%
ComBatLS vs ComBat w/o Covariates		
ComBat w/o Covariates	156	0.75%
ComBatLS	20634	99.20%
ComBatLS vs ComBat-GAM		
ComBat-GAM	1617	7.77%
ComBatLS	18992	91.31%
Linear ComBat vs ComBat w/o Covariates		
ComBat w/o Covariates	189	0.91%
Linear ComBat	20599	99.03%
Linear ComBat vs ComBat-GAM		
ComBat-GAM	7639	36.73%
Linear ComBat	10398	49.99%
Linear ComBat vs ComBatLS		
ComBatLS	19030	91.49%
Linear ComBat	1580	7.60%

Supplemental Table 2. Pairwise tests of absolute z-score errors within each of 208 brain features replicated across 100 subject resamplings. All tests conducted as pairwise, two-tailed t-tests of ranks with Welch's correction. FDR-corrected across 1248 tests (208 features x 6 ComBat method pairings) within each sampling permutation.

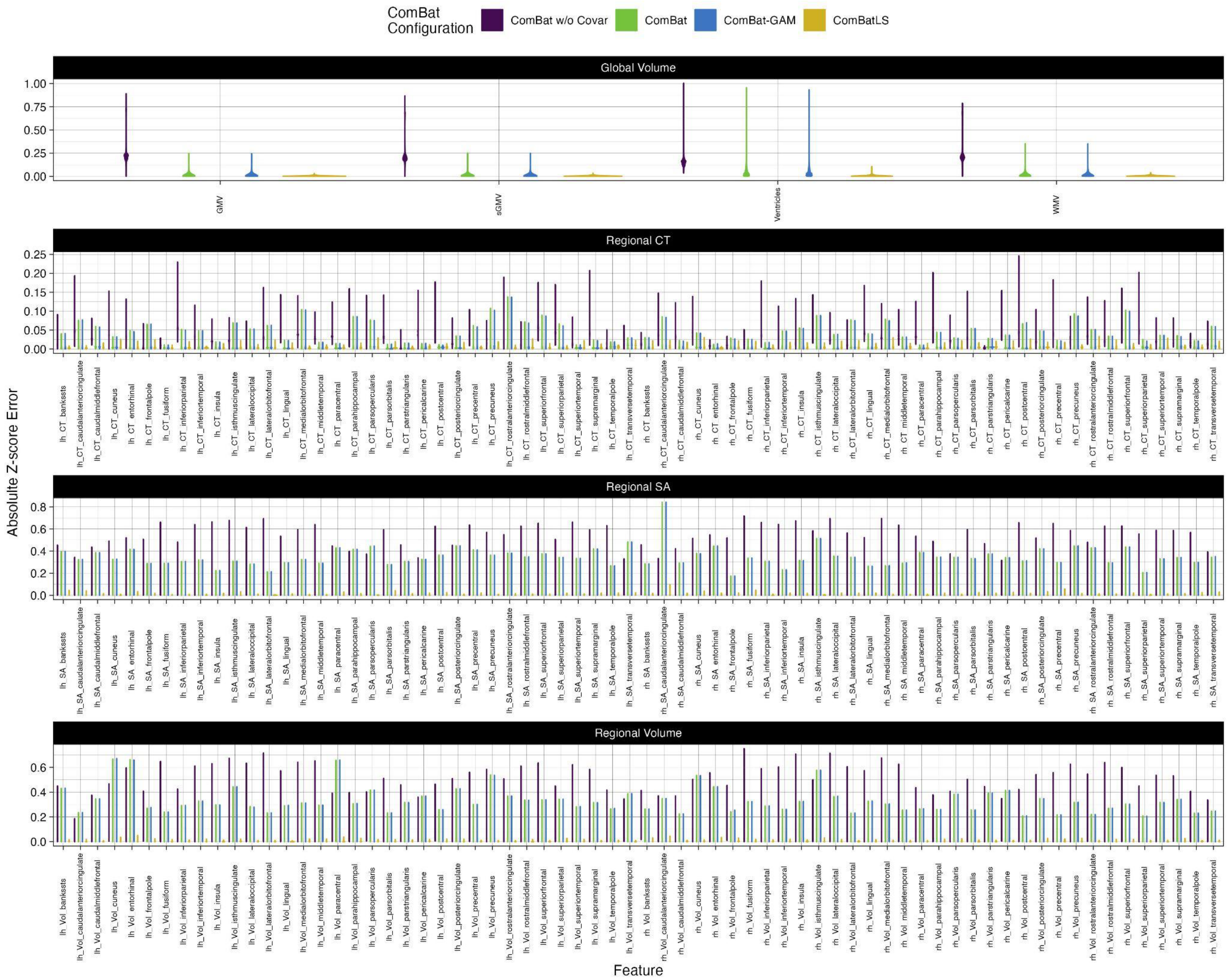

Supplemental Figure 18. Absolute z-score errors across brain features and ComBat methods. Violin plots of absolute z-score errors across 208 brain features. Abbrv: CT, cortical thickness; SA, surface area.

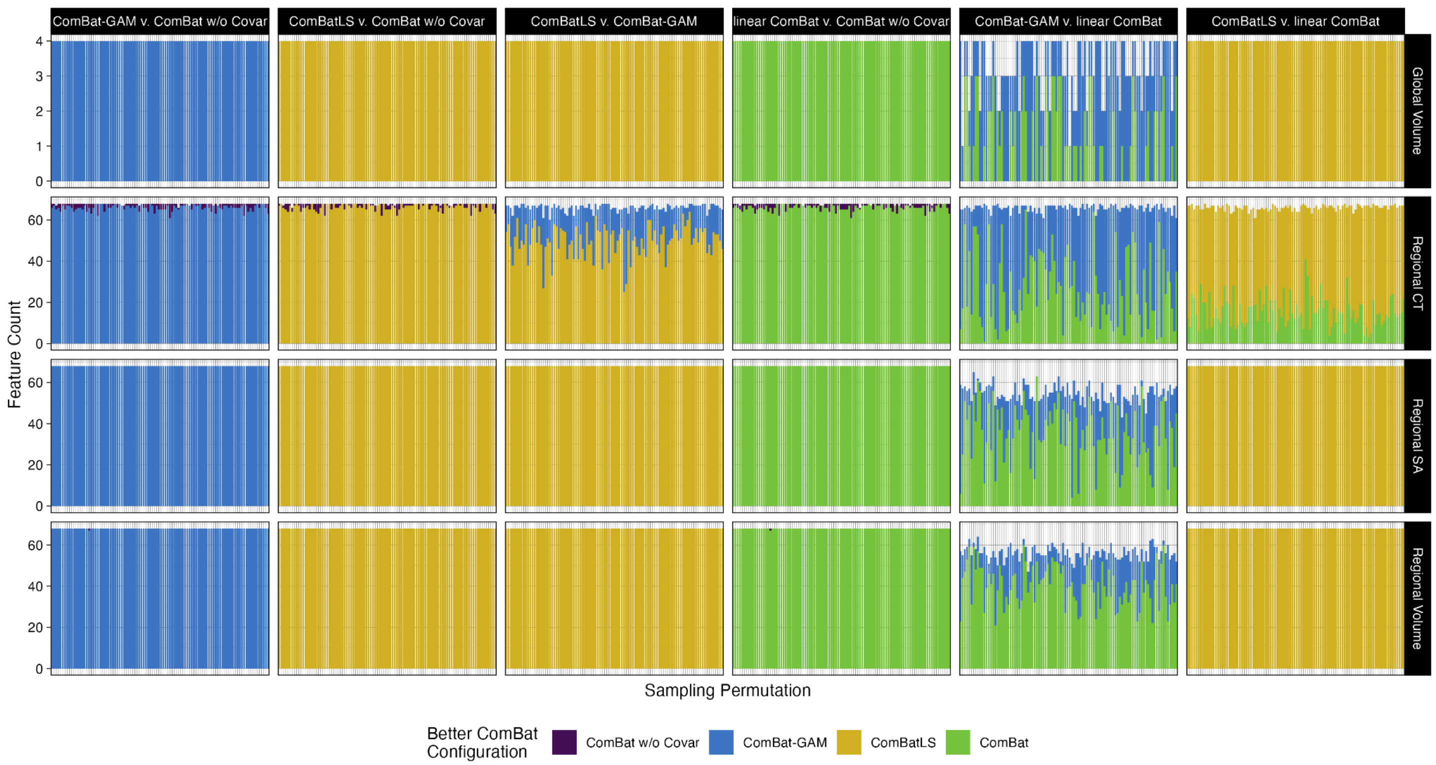

Supplemental Figure 19. Pairwise comparisons of absolute z-score errors across ComBat methods within each brain feature across 100 sampling replications. Fill indicates the ComBat method that produces significantly smaller absolute z-score errors, FDR-corrected across 1248 tests (208 features x 6 ComBat method pairings) within each permutation.

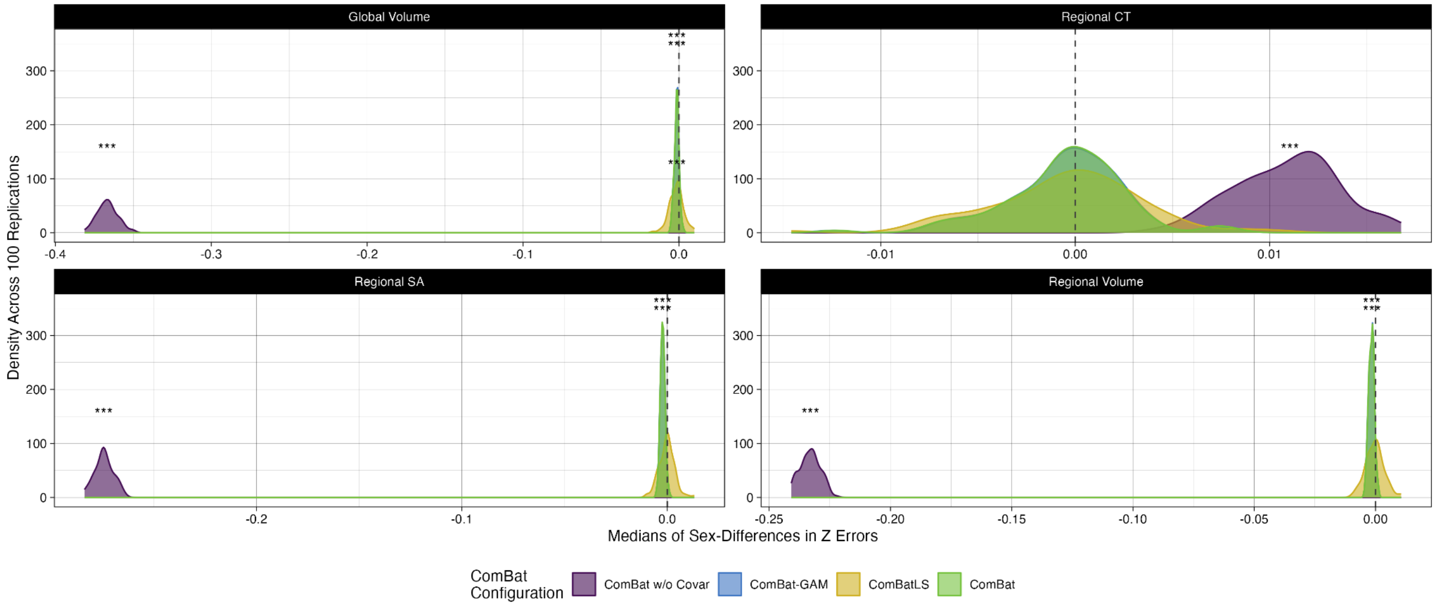

Supplemental Figure 20. Density plots of median sex differences in z-score errors induced by different ComBat methods within phenotype categories across 100 replications. Results are consistent when applying FDR correction across 4 phenotype categories and 4 harmonization methods. Abbrv: CT, cortical thickness; SA, surface area; ***, $p < 0.001$; **, $p < 0.01$.

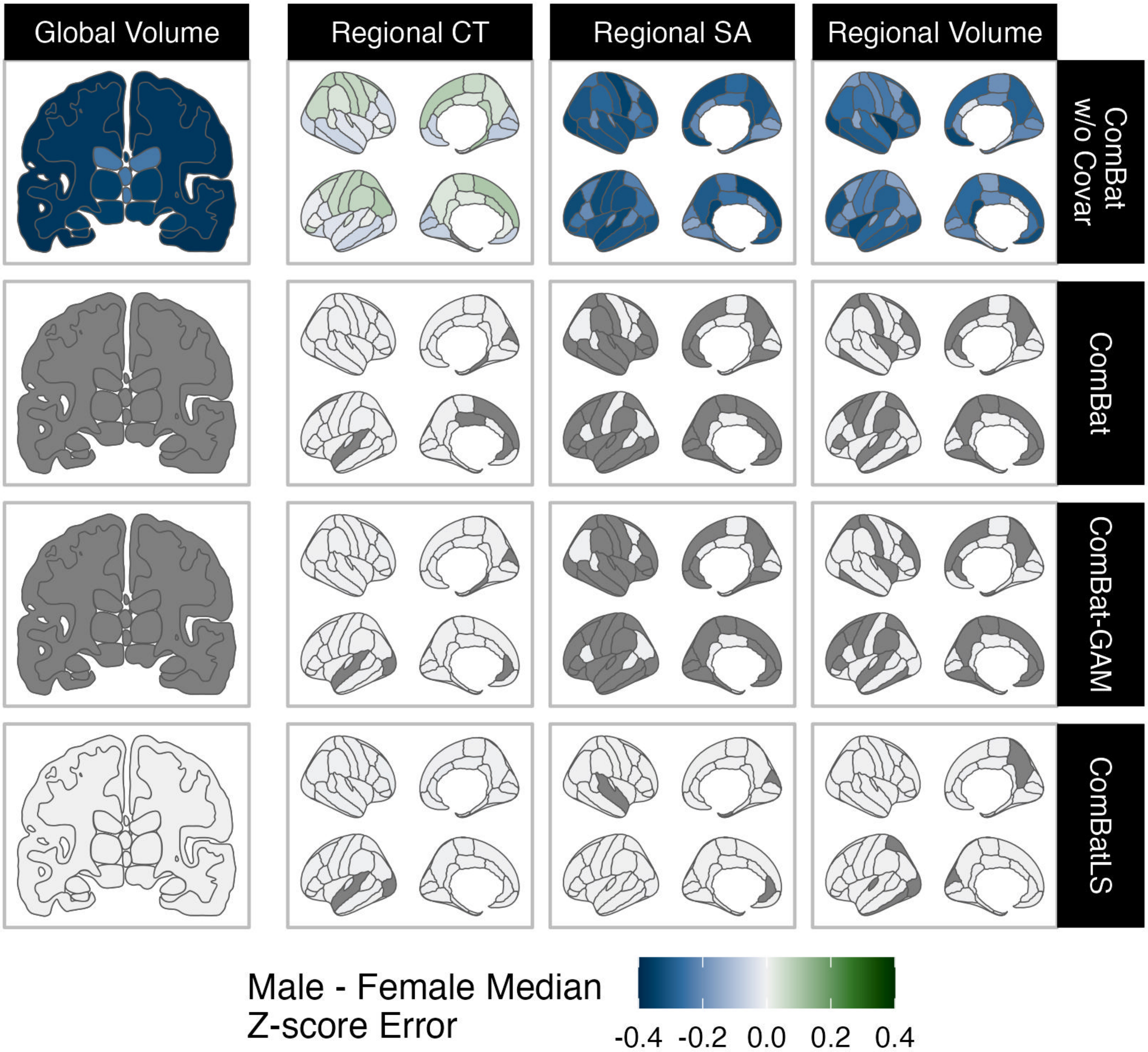

Supplemental Figure 21. Significant differences in males' and females' median z-score errors across brain features and ComBat methods. Positive centile errors (green) indicate that males' z-scores tend to be overestimated relative to females'. Results are consistent when applying FDR correction across regions. Abbrv: CT, cortical thickness; SA, surface area.

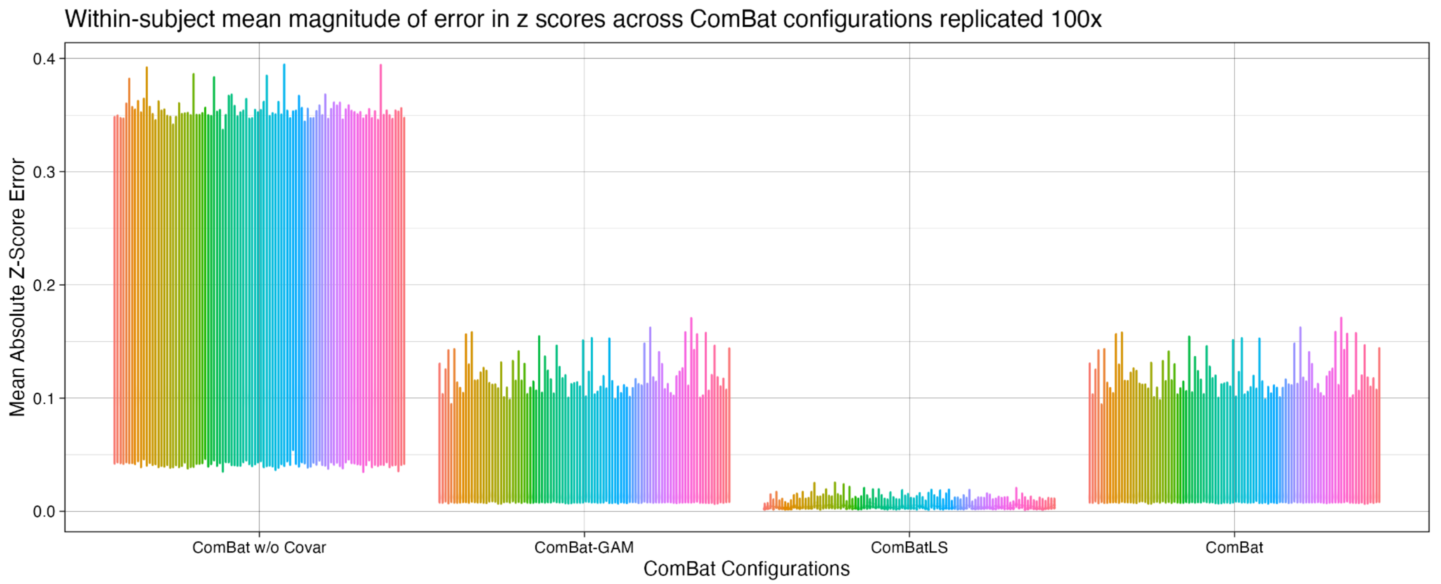

Supplemental Figure 22. Subjects' mean absolute z-score error across ComBat configurations and 100 sampling replications. Violin plots of absolute z-score error for 208 features averaged within subject. Fill corresponds to sampling replication.

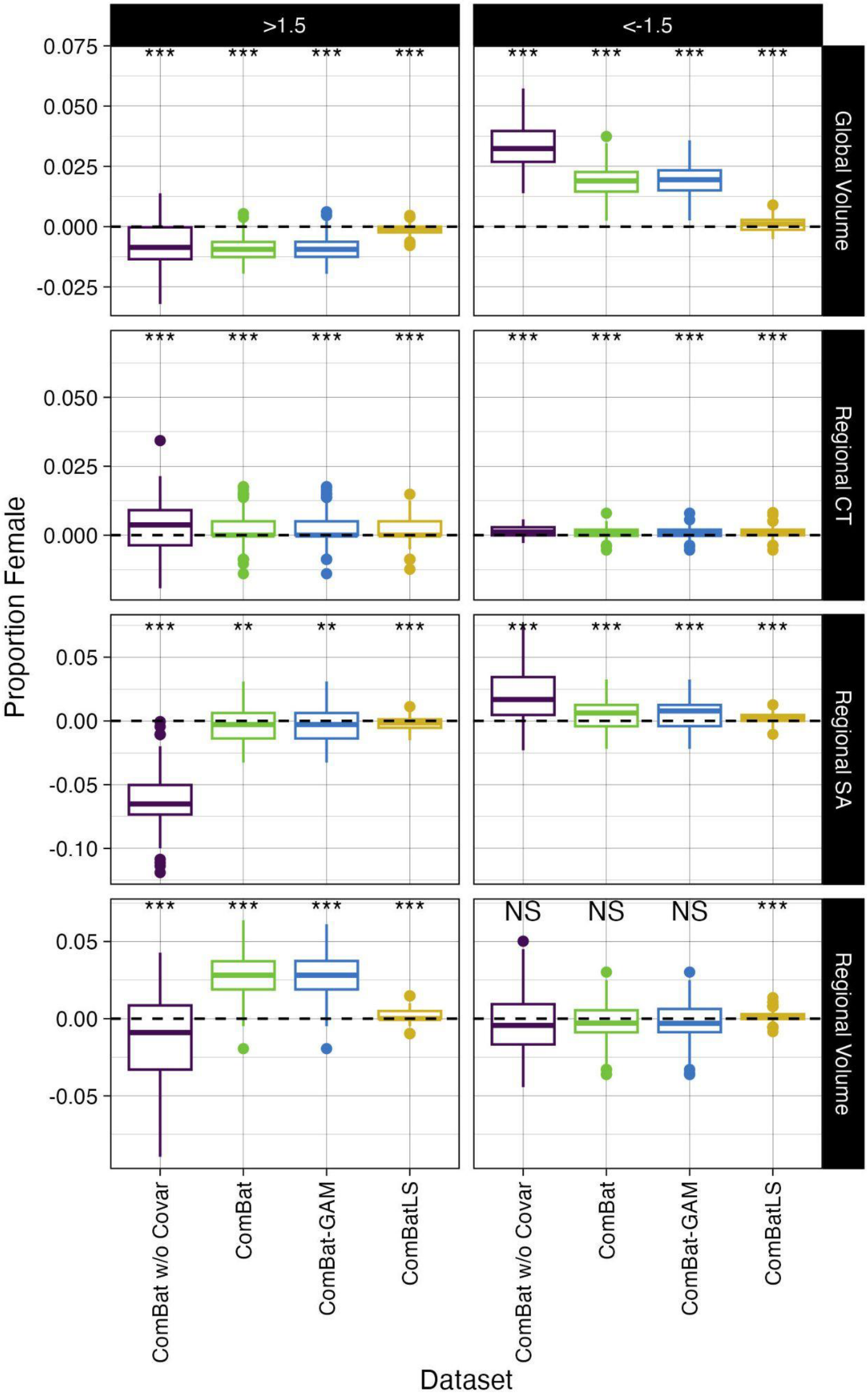

Supplemental Figure 23. Over- or under-representation of females among individuals with extreme z-scores across ComBat methods. Bias in the proportion of females with low (<-1.5) or high (>1.5) mean z-scores across 100 sampling replications. Positive values indicate a higher proportion of females than "true" mean z-scores calculated from unharmonized data (dashed line). Results are consistent when applying FDR correction. Abbrv: ***, p < 0.001; **, p < 0.01.

B) Varying M:F ratios

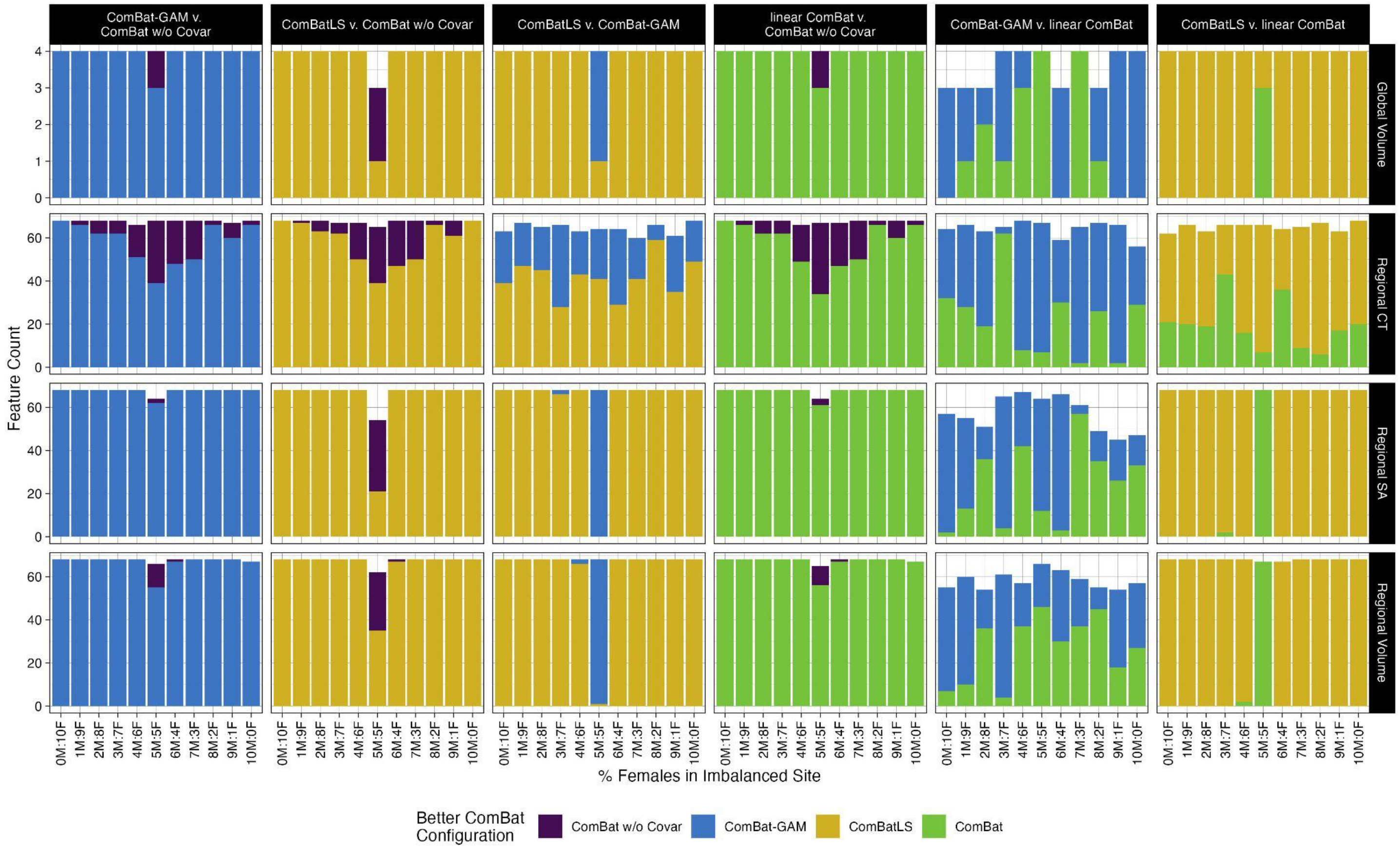

Supplemental Figure 24. Magnitude z-score errors compared pairwise between ComBat methods across varying levels of sex-imbalances in simulated sites. Fill indicates ComBat method with significantly lower absolute z-score errors for a given feature, FDR-corrected across simulated sex ratios. Abbrv: CT, cortical thickness; SA, surface area.

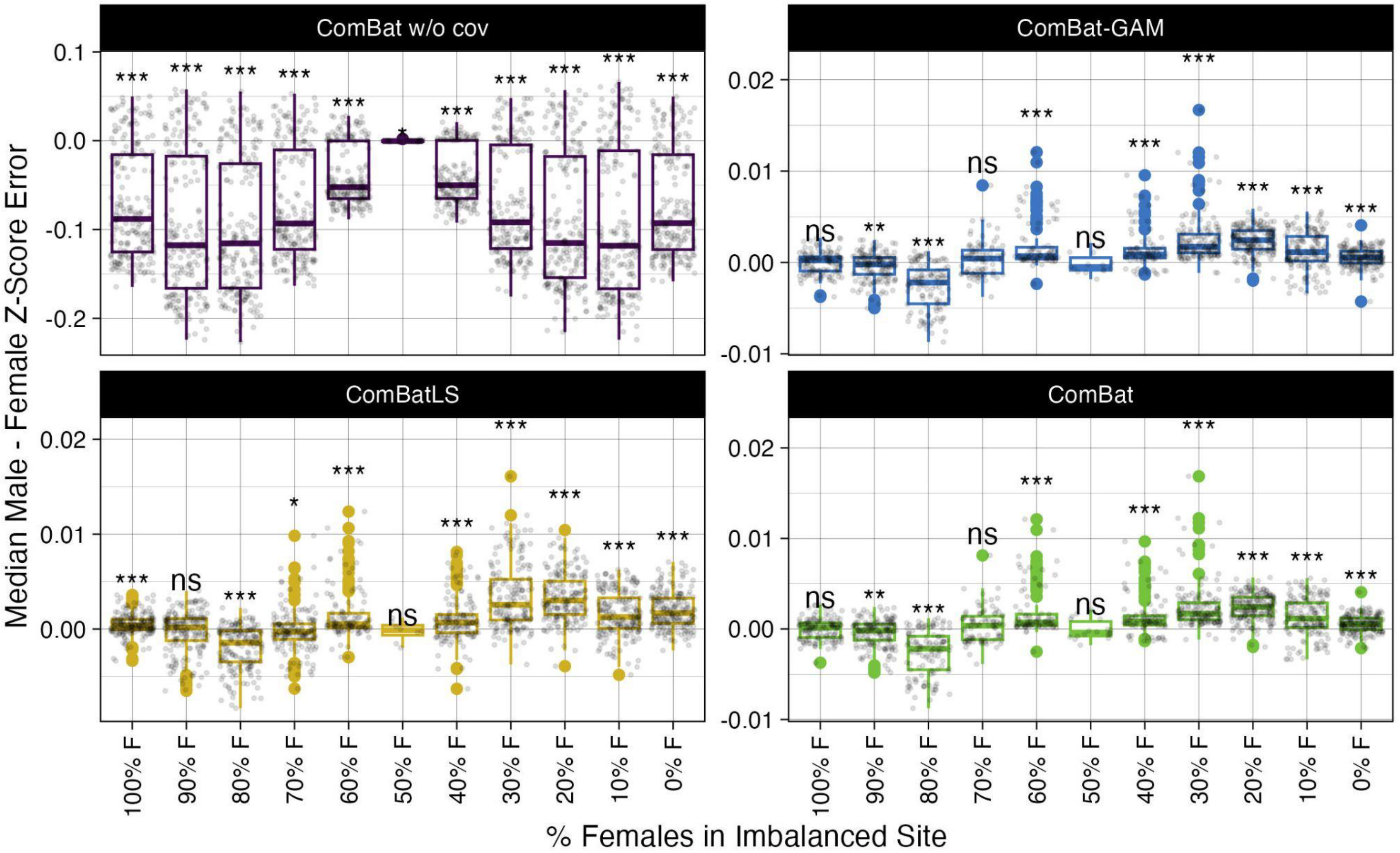

Supplemental Figure 25. Sex-biases in z-score errors induced by various ComBat methods across varying degrees of sex-imbalance. Points show brain features with significant differences in the distributions of males' and females' z-score errors (FDR corrected). Boxplots show median male - median female z-score errors across these features when centiles are derived from data harmonized by different ComBat methods. ComBat without covariate preservation induces strong biases wherein males' z-scores are underestimated relative to females', particularly as simulated sites become more imbalance for sex. Results are highly similar when applying FDR correction for 11 M:F ratios. Abbrv: ***, p < 0.001; **, p < 0.01.

C) Without Extreme Z-scores

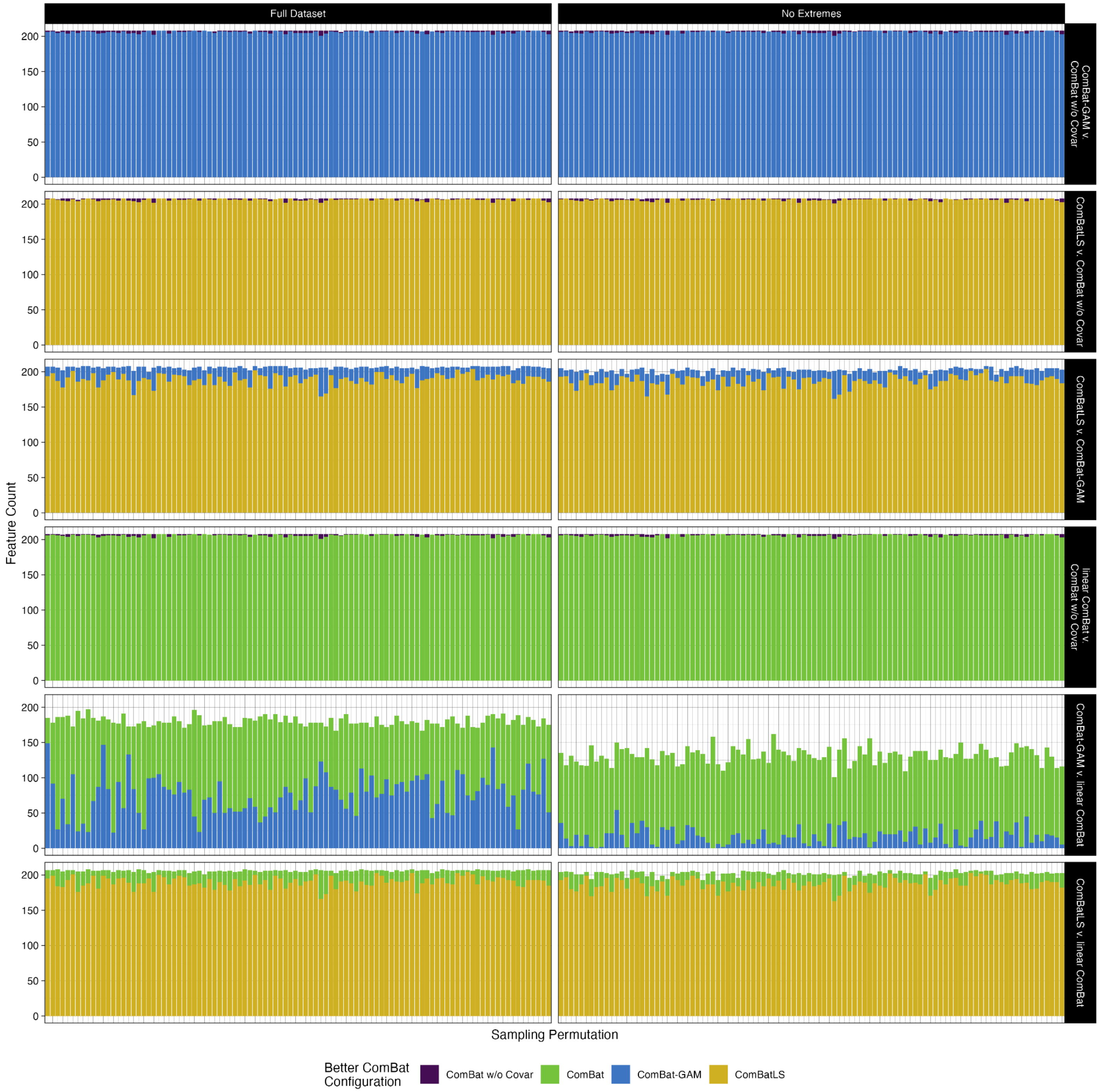

Supplemental Figure 26. Extreme phenotypes do not drive differences in absolute z-score errors between ComBat methods. Comparison of pairwise tests of absolute z-score errors between ComBat methods when z-scores with raw values above 2 or below -2 are excluded. Absolute z-score errors were compared within each brain feature across 100 sampling permutations. Fill indicates the ComBat method that produces significantly smaller absolute errors, FDR-corrected across 1248 tests (208 features x 6 ComBat method pairings) within each permutation.

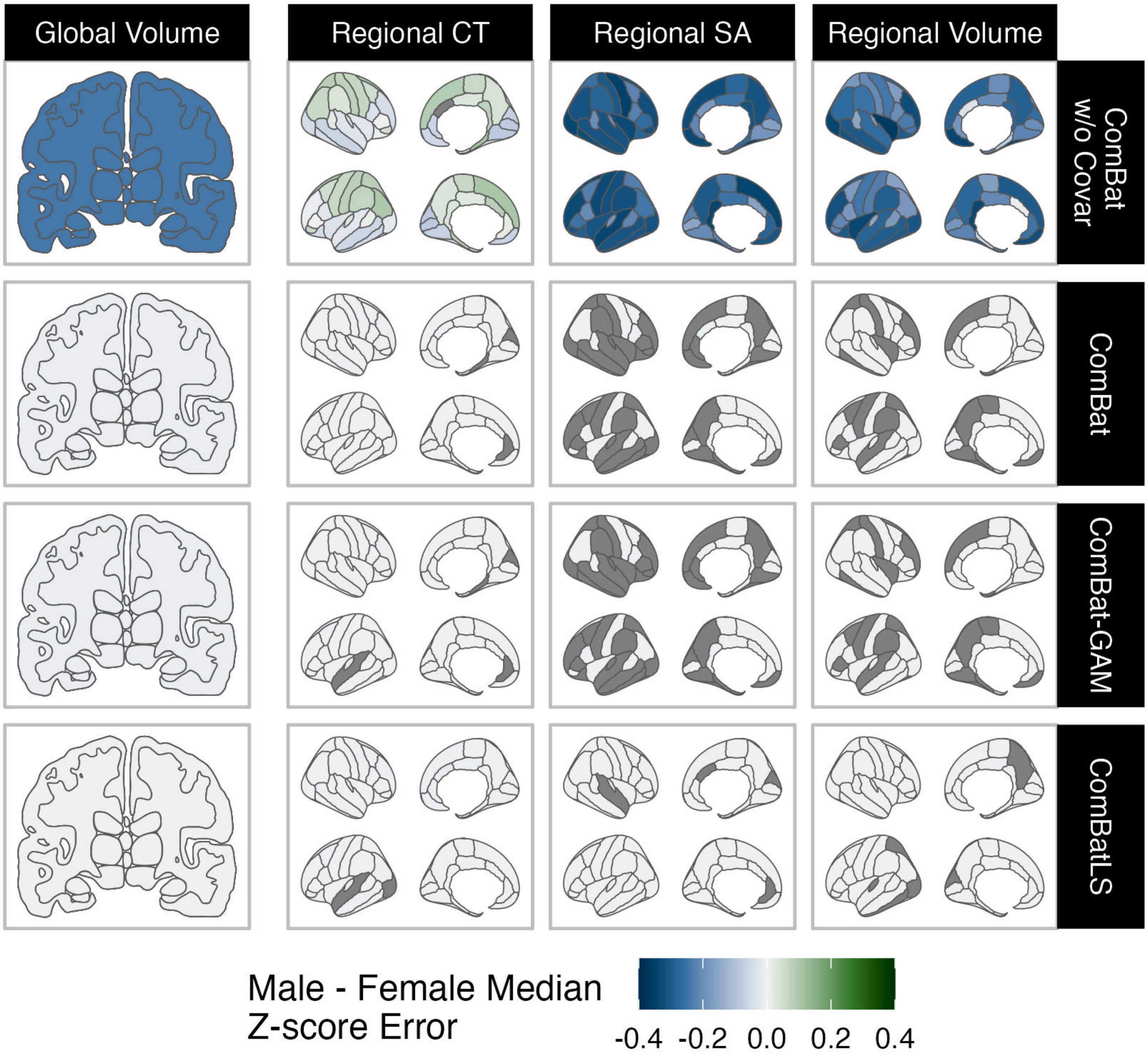

Supplemental Figure 27. Significant differences in males' and females' median z-score errors across brain features and ComBat methods when extreme features are excluded. Positive z-score errors (green) indicate that males' z-score tend to be overestimated relative to females'. Results are consistent when applying FDR correction across features. Abbrv: CT, cortical thickness; SA, surface area.

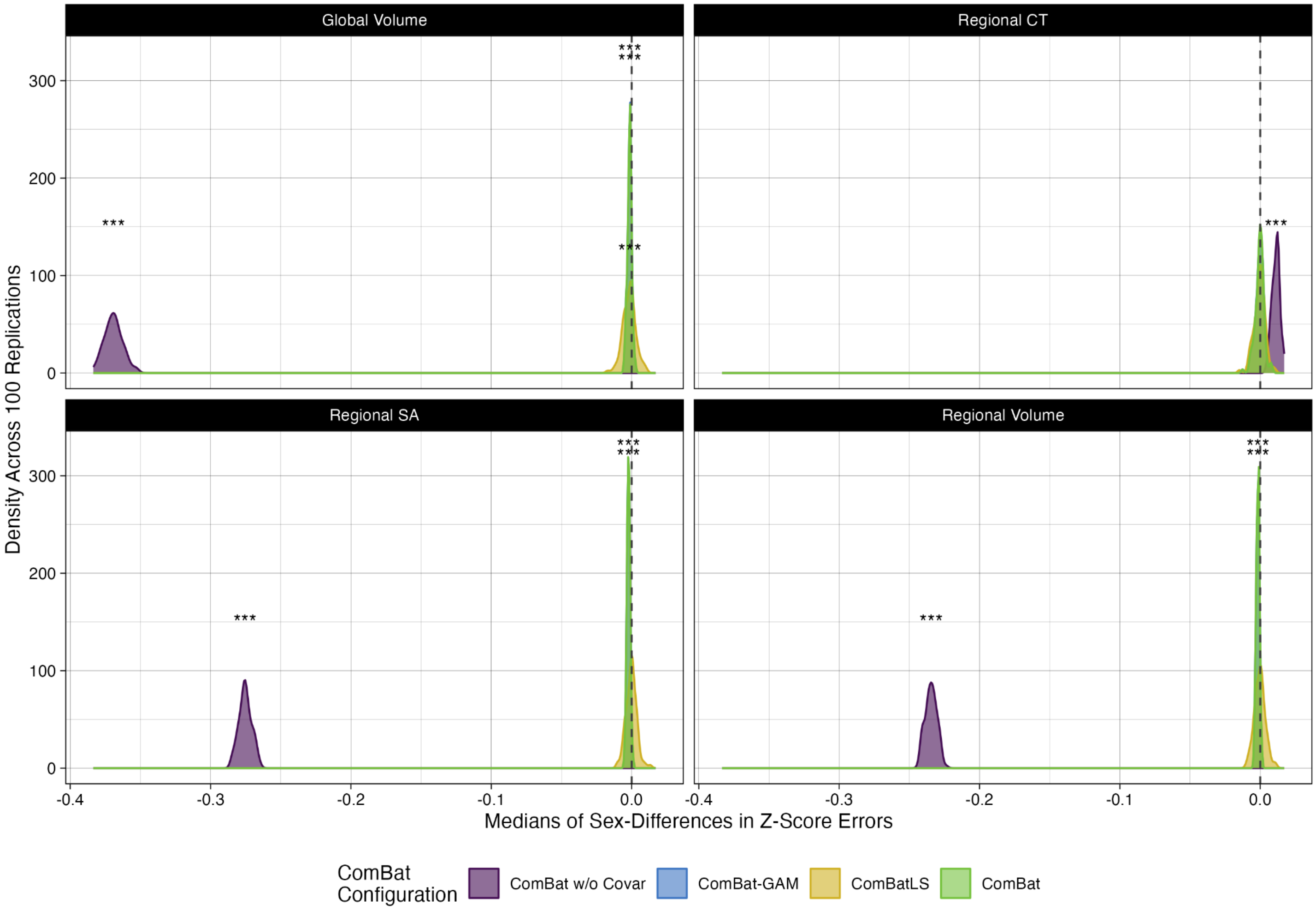

Supplemental Figure 28. Density plots of median sex differences in z-score errors induced by different ComBat methods across 100 replications when extreme phenotypes are excluded. Results are consistent when applying FDR correction across 4 phenotype classes and 4 harmonization methods. Abbrv: CT, cortical thickness; SA, surface area; ***, p < 0.001; **, p < 0.01.

<u>Section VI. Comparisons of ComBatLS and ComBat-GAM for harmonizing consortium data</u>

A) Main Results

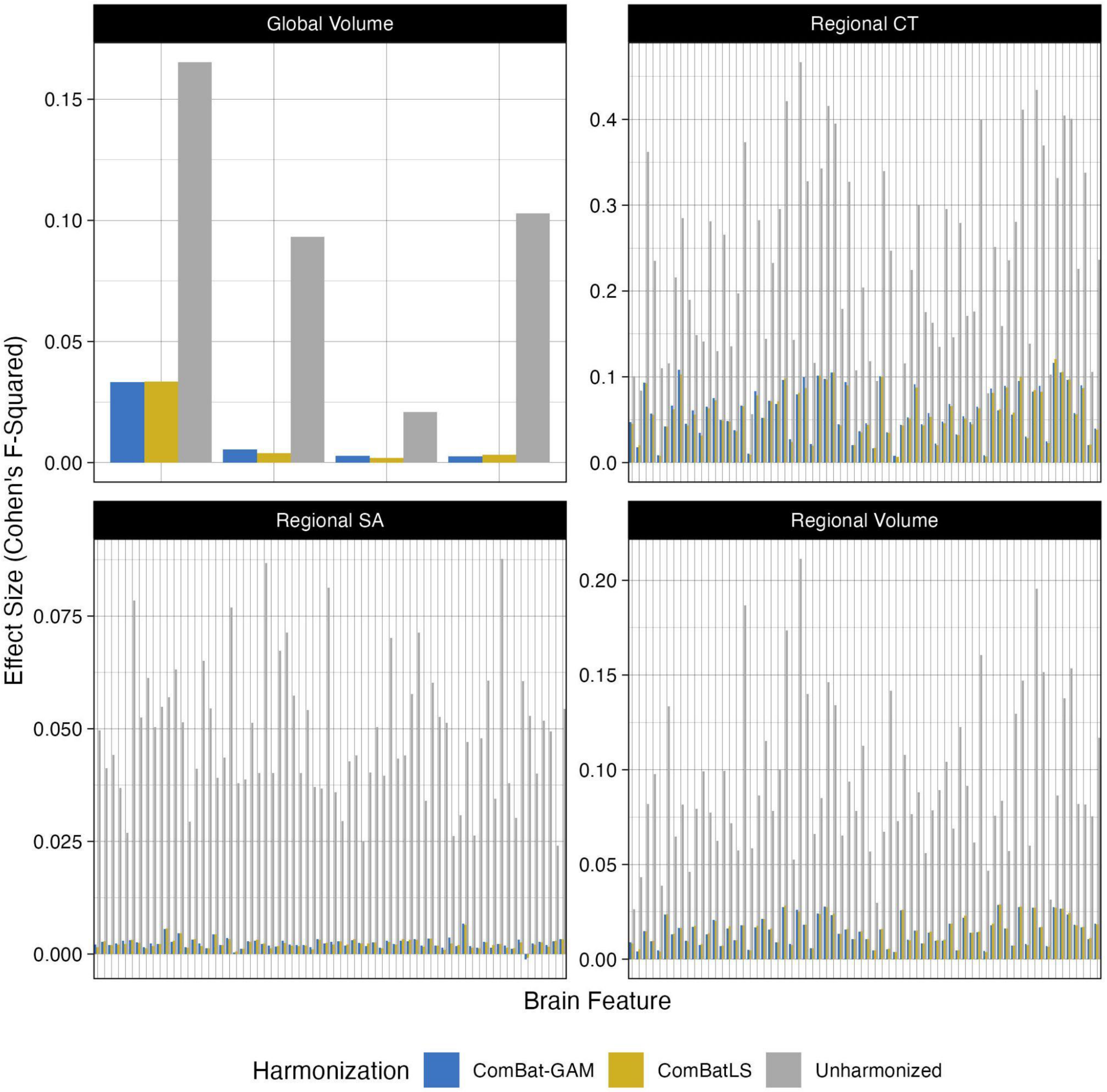

Supplemental Figure 29. Residual effects of study following harmonization with ComBatLS or ComBat-GAM relative to unharmonized data. Effect size for study in each brain feature's gamlss growth chart after harmonization. Abbrv: CT, cortical thickness; SA, surface area.

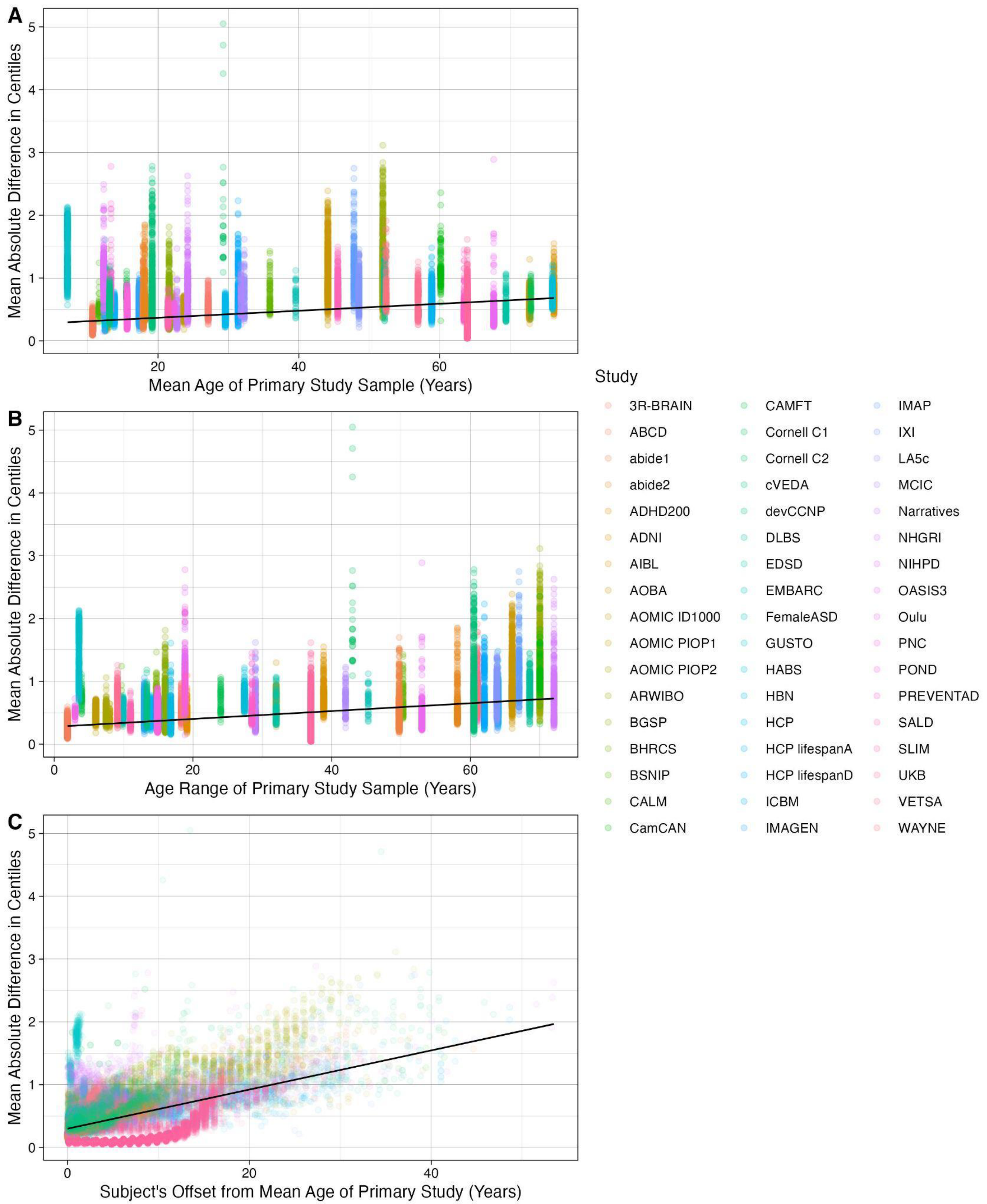

Supplemental Figure 30. Absolute differences in ComBatLS and ComBat-GAM-derived centiles are

related to age's distribution across batches. A) The mean age of a study's subjects is significantly associated with the magnitude difference in their centile scores, such that centiles for individuals from older studies tend to be more impacted by the choice of harmonization method (Beta=0.0056 centiles, $p < 0.001$). B) Absolute differences in centile scores are also associated with the range of ages included in a batch's sample, with subjects from studies with broader age ranges being more impacted by harmonization method (Beta=0.0063 centiles, $p < 0.001$). C) Finally, how greatly any given individual's centiles differ when using ComBatLS or ComBat-GAM depends on the magnitude of difference between their age and that of their study sample's mean, with subjects who are less well-described by their study's mean age being impacted most greatly (Beta=0.031, $p < 0.001$). Y-axes shows mean absolute difference in a subject's centile scores across brain features when derived from ComBatLS- or ComBat-GAM-harmonized data. Each point represents one individual while fill represents primary study. All trend lines indicate marginal effects for study's sample size. Gray bands (not visible) represents 95% confidence interval of marginal association.

B) Validating generalizability to other normative models

We designed ComBatLS with normative modeling in mind, as these models and their resulting scores 1) depend strongly on their input data accurately representing phenotypes' distributions across a population and 2) require vast enough datasets that some form of harmonization across batches is almost inevitable. However, there are numerous statistical approaches to fitting normative models (see (Borghi et al., 2006; Ge et al., 2024).Thus, we sought to validate that the benefits we observed in ComBatLS on normative scores generalized to other methods for model fitting that also require data be harmonzied. We therefore borrowed the approach popularized by (Frangou et al., 2022). As with our primary analyses in the LBCC, we fit these models on ComBat-GAM and ComBatLS-harmonized data, then assessed 1) how well each method was able to harmonize the dataset, as evidenced by low residual site effects in the derived models, and 2) whether differences in ComBat-GAM and ComBatLS-derived normative scores are related to batch demographics, which suggests ComBatLS may improve the accuracy of such scores.

Using the same LBCC data which was harmonized using ComBat-GAM and ComBatLS (see Section 2.3 of the main text), we fit normative models covering ages 3 to 90 years for each brain phenotype using the LMS method (Cole & Green, 1992) as in (Dima et al., 2021; Frangou et al., 2022) and implemented at https://centilebrain.org/#/tutorial. Notably, this approach requires that models are fit separately in each sex, with the only covariate being a smooth effect of age. As above, we also refit these models with additional 'study' term, which we used to estimate residual batch effects in each dataset. As with our main analyses, we also obtained centile scores across every phenotype, which we used to determine changes in whether subjects were classified as "extreme" and the average differences in each subjects' ComBat-GAM and ComBatLS-derived centiles. Finally, we tested whether subjects' average absolute centile differences were related to their batch's mean age, age range, or the offset from the batch's mean age.

As in our main analyses, we found that ComBatLS and ComBat-GAM mitigated batch effects comparably across all features with small residual study effects in the resultant normative models (Cohen's F-squared: ComBatLS median=0.013, IQR=0.012; ComBat-GAM median=0.012, IQR=0.012; Unharmonized median = 0.200, IQR = 0.33; **SFig 31A**). We again found differences in ComBatLS or ComBat-GAM-derived centiles (mean absolute difference in centile scores = 0.443, range = 0.06 - 7.16 centiles) with 51.7% of subjects having discrepant categorization of extremely high (< 5%) or low (> 95%) centiles in at least one feature (mean=0.954 features per subject, range = 0 - 40 features). Finally, as in our exploratory analyses in the main text, we found that subjects' ComBatLS- and ComBat-GAM-harmonized centile scores varied with the mean

age of the study sample (Beta=0.005, $p<0.001$; **SFig 31B**), the range of ages included in a sample (Beta=0.006, $p<0.001$; **SFig 31 C**), and how much an individuals' age deviated from that sample's mean age (Beta=0.032, $p<0.0001$; **SFig 31 D**) when controlling for the sample's size.

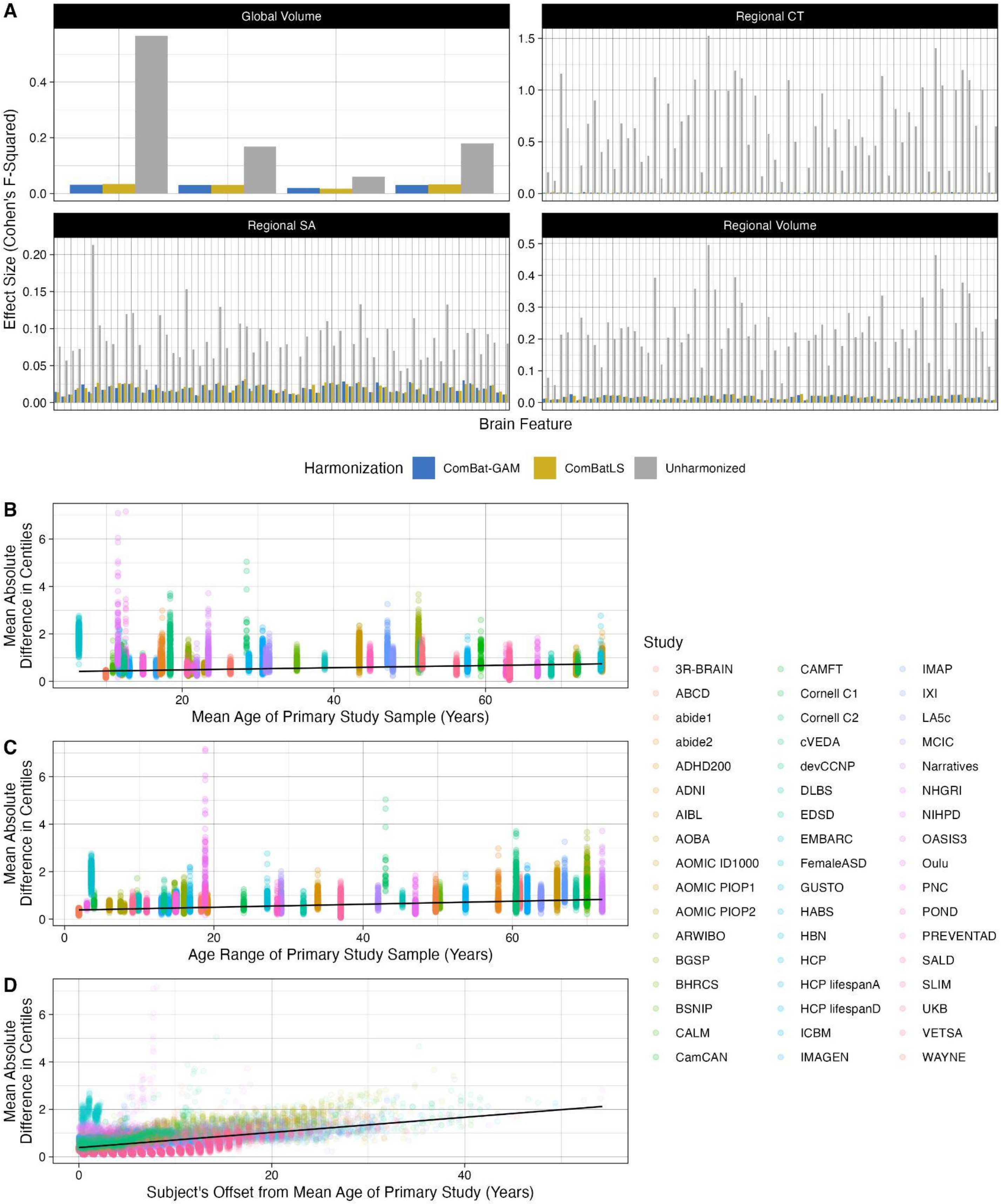
A
Global Volume
Regional CT
Regional SA
Regional Volume
Effect Size (Cohen's F-Squared)
Brain Feature
Harmonization
ComBat-GAM
ComBatLS
Unharmonized
B
Mean Absolute Difference in Centiles
Mean Age of Primary Study Sample (Years)
C
Age Range of Primary Study Sample (Years)
D
Subject's Offset from Mean Age of Primary Study (Years)
Study
3R-BRAIN
ABCD
abide1
abide2
ADHD200
ADNI
AIBL
AOBA
AOMIC ID1000
AOMIC PIOP1
AOMIC PIOP2
ARWIBO
BGSP
BHRCS
BSNIP
CALM
CamCAN
CAMFT
Cornell C1
Cornell C2
cVEDA
devCCNP
DLBS
EDSD
EMBARC
FemaleASD
GUSTO
HABS
HBN
HCP
HCP lifespanA
HCP lifespanD
ICBM
IMAGEN
IMAP
IXI
LA5c
MCIC
Narratives
NHGRI
NIHPD
OASIS3
Oulu
PNC
POND
PREVENTAD
SALD
SLIM
UKB
VETSA
WAYNE

Supplemental Figure 31. ComBatLS's performance generalizes to alternate normative modeling methods. A) Effect size for study in each brain feature's LMS growth chart after harmonization. B) Abbrv: CT, cortical thickness; SA, surface area. B-D) Absolute differences in ComBatLS and ComBat-GAM-derived centiles vary with age's distribution across batches when controlling for batch size, showing positive relationships with (B) studies' mean ages (Beta=0.005, $p<0.001$), (C) studies' age ranges (Beta=0.006, $p<0.001$), and (D) the offset between a subject and their study's mean age (Beta=0.032, $p<0.0001$). Each point represents one individual while fill represents primary study. All trend lines indicate marginal effects analyses controlling for study sample size. Gray bands (not visible) represent 95% confidence interval of marginal association.

Section VII. References

Borghi, E., de Onis, M., Garza, C., Van den Broeck, J., Frongillo, E. A., Grummer-Strawn, L., Van Buuren, S., Pan, H., Molinari, L., Martorell, R., Onyango, A. W., Martines, J. C., Pinol, A., Siyam, A., Victoria, C. G., Bhan, M. K., Araújo, C. L., Lartey, A., Owusu, W. B., … Heinig, M. J. (2006). Construction of the World Health Organization child growth standards: Selection of methods for attained growth curves. *Statistics in Medicine*, *25*(2), 247–265. https://doi.org/10.1002/SIM.2227

Cole, T. J., & Green, P. J. (1992). Smoothing reference centile curves: The lms method and penalized likelihood. *Statistics in Medicine*, *11*(10), 1305–1319. https://doi.org/10.1002/sim.4780111005

Dima, D., Modabbernia, A., Papachristou, E., Doucet, G. E., Agartz, I., Aghajani, M., Akudjedu, T. N., Albajes-Eizagirre, A., Alnæs, D., Alpert, K. I., Andersson, M., Andreasen, N. C., Andreassen, O. A., Asherson, P., Banaschewski, T., Bargallo, N., Baumeister, S., Baur-Streubel, R., Bertolino, A., … Frangou, S. (2021). Subcortical volumes across the lifespan: Data from 18,605 healthy individuals aged 3–90 years. *Human Brain Mapping*, *43*(1), 452–469. https://doi.org/10.1002/hbm.25320

Frangou, S., Modabbernia, A., Williams, S. C. R., Papachristou, E., Doucet, G. E., Agartz, I., Aghajani, M., Akudjedu, T. N., Albajes-Eizagirre, A., Alnaes, D., Alpert, K. I., Andersson, M., Andreasen, N. C., Andreassen, O. A., Asherson, P., Banaschewski, T., Bargallo, N., Baumeister, S., Baur-Streubel, R., … Dima, D. (2022). Cortical thickness across the lifespan: Data from 17,075 healthy individuals aged 3-90 years. *Human Brain Mapping*, *43*(1), 431–451. https://doi.org/10.1002/hbm.25364

Ge, R., Yu, Y., Qi, Y. X., Fan, Y., Chen, S., Gao, C., Haas, S. S., New, F., Boomsma, D. I., Brodaty, H., Brouwer, R. M., Buckner, R., Caseras, X., Crivello, F., Crone, E. A., Erk, S., Fisher, S. E., Franke, B., Glahn, D. C., … Yu, K. (2024). Normative modelling of brain morphometry across the lifespan with CentileBrain: Algorithm benchmarking and model optimisation. *The Lancet Digital Health*, *6*(3), e211–e221. https://doi.org/10.1016/S2589-7500(23)00250-9
